# Exploring the cell-free total RNA transcriptome in diffuse large B-cell lymphoma and primary mediastinal B-cell lymphoma patients as biomarker source in blood plasma liquid biopsies

**DOI:** 10.3389/fonc.2023.1221471

**Published:** 2023-10-25

**Authors:** Philippe Decruyenaere, Edoardo Giuili, Kimberly Verniers, Jasper Anckaert, Katrien De Grove, Malaïka Van der Linden, Dries Deeren, Jo Van Dorpe, Fritz Offner, Jo Vandesompele

**Affiliations:** ^1^ Department of Hematology, Ghent University Hospital, Ghent, Belgium; ^2^ OncoRNALab, Cancer Research Institute Ghent (CRIG), Ghent University, Ghent, Belgium; ^3^ Department of Biomolecular Medicine, Ghent University, Ghent, Belgium; ^4^ Interuniversity Institute of Bioinformatics in Brussels (IB^2^), Free University of Brussels, Brussels, Belgium; ^5^ Department of Biotechnology and Pharmacy, University of Bologna, Bologna, Italy; ^6^ Department of Pathology, Ghent University Hospital, Ghent, Belgium; ^7^ Department of Hematology, Algemeen Ziekenhuis (AZ) Delta Roeselare-Menen, Roeselare, Belgium

**Keywords:** cell-free RNA, liquid biopsy, blood plasma, biomarkers, DLBCL, diffuse large B-cell lymphoma, PMBCL, primary mediastinal B-cell lymphoma

## Abstract

**Introduction:**

Diffuse large B-cell lymphoma (DLBCL) and primary mediastinal B-cell lymphoma (PMBCL) are aggressive histological subtypes of non-Hodgkin’s lymphoma. Improved understanding of the underlying molecular pathogenesis has led to new classification and risk stratification tools, including the development of cell-free biomarkers through liquid biopsies. The goal of this study was to investigate cell-free RNA (cfRNA) biomarkers in DLBCL and PMBCL patients.

**Materials and methods:**

Blood plasma samples (n=168) and matched diagnostic formalin-fixed paraffin-embedded (FFPE) tissue samples (n=69) of DLBCL patients, PMBCL patients and healthy controls were collected between 2016-2021. Plasma samples were collected at diagnosis, at interim evaluation, after treatment, and in case of refractory or relapsed disease. RNA was extracted from 200 µl plasma using the miRNeasy serum/plasma kit and from FFPE tissue using the miRNeasy FFPE kit. RNA was subsequently sequenced on a NovaSeq 6000 instrument using the SMARTer Stranded Total RNA-seq pico v3 library preparation kit.

**Results:**

Higher cfRNA concentrations were demonstrated in lymphoma patients compared to healthy controls. A large number of differentially abundant genes were identified between the cell-free transcriptomes of DLBCL patients, PMBCL patients, and healthy controls. Overlap analyses with matched FFPE samples showed that blood plasma has a unique transcriptomic profile that significantly differs from that of the tumor tissue. As a good concordance between tissue-derived gene expression and the immunohistochemistry Hans algorithm for cell-of-origin (COO) classification was demonstrated in the FFPE samples, but not in the plasma samples, a 64-gene cfRNA classifier was developed that can accurately determine COO in plasma. High plasma levels of a 9-gene signature (*BECN1*, *PRKCB*, *COPA*, *TSC22D3*, *MAP2K3*, *UQCRHL*, *PTMAP4*, *EHD1*, *NAP1L1* pseudogene) and a 5-gene signature (*FTH1P7*, *PTMAP4*, *ATF4*, *FTH1P8*, *ARMC7*) were significantly associated with inferior progression-free and overall survival in DLBCL patients, respectively, independent of the NCCN-IPI score.

**Conclusion:**

Total RNA sequencing of blood plasma samples allows the analysis of the cell-free transcriptome in DLBCL and PMBCL patients and demonstrates its unexplored potential in identifying diagnostic, cell-of-origin, and prognostic cfRNA biomarkers.

## 1 Introduction

Diffuse large B-cell lymphoma (DLBCL) is the most common histological subtype of non-Hodgkin’s lymphomas (NHL), representing approximately 25% of new diagnoses. DLBCL can occur *de novo* or because of transformation from low-grade B-cell lymphomas (1). Although a sustained complete response (CR) can be obtained in most patients with first-line R-CHOP immunochemotherapy (rituximab, cyclophosphamide, vincristine, doxorubicin, and prednisone), patients with refractory or relapsed (R/R) disease have a poor prognosis, despite second-line treatments (2).

Two decades ago, gene expression profiling (GEP) was used to discriminate between different cell-of-origin (COO) DLBCL subgroups, germinal center B-cell–like (GCB) and activated B-cell–like (ABC), which was clinically implemented through the use of surrogate immunohistochemistry (IHC) algorithms (3, 4). Since then, DLBCL has increasingly been recognized as a highly heterogeneous disease with respect to morphology, genetics, and biological behavior. This evolution was exemplified by recognizing primary mediastinal B-cell lymphoma (PMBCL) as a separate entity by the World Health Organization (WHO) in 2008 based on clinicopathologic features and a distinct molecular signature that overlapped with nodular sclerosis classical Hodgkin lymphoma (5–7). PMBCL accounts for 2-4% of NHL, and although outcomes have improved in the modern rituximab era, standard treatment is still a topic of debate and refractory disease correlates with poor outcome. Moreover, approximately 5% of DLBCL patients show a molecular PMBCL phenotype in the absence of mediastinal involvement, demonstrating the overlap, complexity, and challenges of current classifications (8). More recent evolutions include the identification of the double expressor (DEL) DLBCL subtype, characterized by an overexpression of MYC and BCL2 proteins not related to underlying chromosomal rearrangement, and double-hit lymphomas (DHL), a separate entity defined as a dual rearrangement of MYC and BCL2 and/or BCL6, both associated with poor prognosis (1, 9). The increasing number of subtypes highlights that, although major progress has been made, further in-depth molecular analyses are needed to identify and characterize high-risk patients, to elucidate the biological pathways involved and reveal therapeutic targets, and to ultimately improve outcomes. In this setting, profiling through massively parallel sequencing has proven to be a powerful tool.

A liquid biopsy is the process of investigating tumor-derived cells or biomaterials like cell-free nucleic acids, metabolites, proteins, or extracellular vesicles through biofluid sampling, without the need of a tissue biopsy. Potential advantages include its non-invasive nature, its ability to reflect inter- and intra-tumor heterogeneity, and the possibility of repeated measurements through longitudinal profiling during disease course or treatment (10). Although most studies have focused on the use of cell-free or circulating tumor DNA fragments (cfDNA/ctDNA), there has been increased interest in different forms of coding and non-coding cfRNA, both circulating and extracellular vesicle/platelet-encapsulated, including messenger RNA (mRNA), microRNA (miRNA), long non-coding RNA (lncRNA), and circular RNA (circRNA) (11). Higher circulating transcript levels have been found in patients with solid and hematological malignancies, in which these are considered to play crucial roles in intercellular communication and to contribute to proliferation, malignant transformation, angiogenesis, and immune response escape (12, 13). The goal of this study was to explore the cell-free transcriptome of DLBCL and PMBCL patients using longitudinally collected plasma and matched formalin-fixed paraffin-embedded (FFPE) tissue samples, and to identify diagnostic, COO, and prognostic biomarkers, complementary to current risk stratification tools.

## 2 Materials and methods

### 2.1 Sample collection and processing

#### 2.1.1 Sample collection

A total of 168 longitudinally collected plasma samples derived from 41 DLBCL NOS (hereinafter referred to as DLBCL) and 14 PMBCL patients were collected during the period 06/2016 and 09/2021 at Ghent University Hospital in Ghent (Belgium) and AZ Delta hospital in Roeselare (Belgium). Plasma samples were drawn at the time of diagnosis, at interim evaluation (after 4 cycles of immunochemotherapy), at final evaluation, and in the case of R/R disease. An overview of the collected plasma samples at each timepoint can be found in **Supplementary Figure 1**. Diagnostic FFPE samples were obtained from all patients that had a diagnostic plasma sample, except for 2 DLBCL and 1 PMBCL patient, of which insufficient FFPE material was available for RNA extraction. The study also included plasma samples from 22 healthy controls, as well as 29 FFPE samples derived from non-malignant lymph node tissue. Blood samples were drawn using PAXgene blood ccfDNA tubes (BD Biosciences, 768165) and processed to plasma within 4 hours after blood draw. Immunohistochemical staining was routinely performed on each DLBCL FFPE tissue sample for the markers CD10, BCL6, MUM1, BCL2 and MYC to determine COO classification and double expressor status. Fluorescence *in situ* hybridization (FISH) was performed for MYC and BCL2 rearrangements to identify DHL. Therapy response was assessed by F-fluorodeoxyglucose Positron Emission Tomography/computerized tomography (FDG-PET/CT) according to the Lugano guidelines (14). Response to first-line therapy was defined as obtaining a sustained complete remission (CR) without evidence of relapsed disease within the follow-up period (mean follow-up of 1018 and 1049 days for the DLBCL and PMBCL patients, respectively).

#### 2.1.2 Preparation of plasma and FFPE scrolls

Plasma was obtained by using a one-step centrifugation protocol (1900g x 15min without brake at room temperature) and subsequently frozen and stored at -80°C. FFPE samples were obtained from the Pathology Department of Ghent University Hospital and were re-examined by an experienced pathologist to confirm the correct diagnosis, to determine the percentage of tumor invasion, and to select adequate tumor blocks. Five scrolls of 10 µm were cut from FFPE blocks on a Leica RM2125 RTS manual rotary microtome (Leica Microsystems, Germany) and put into RNase-free Eppendorf tubes (Eppendorf, Germany). If the FFPE also included non-malignant tissue, macrodissection was performed to enrich lymphoma invaded tissue. The FFPE scrolls were processed within 4 hours after preparation.

#### 2.1.3 RNA extraction

RNA extraction was performed on 200 µl of plasma and on 5 scrolls of 10 µm using the miRNeasy serum/plasma kit and miRNeasy FFPE kit, respectively. In each plasma sample, 2 µL of Sequin spike-in controls were added to the sample lysates (1/5,000 of stock solution mix A; Garvan Institute of Medical Research), as well as 2 µL of External RNA Control Consortium (ERCC) spike-in controls (1/12,500; ThermoFisher Scientific, 4456740) were added to the RNA eluate. Genomic DNA was removed by adding 1 μL HL-dsDNase (ArcticZymes, 70800-202) and 1.6 µL reaction buffer (ArcticZymes, 66001) to 12 µL RNA eluate, followed by 10 min incubation at 37°C, and 10 min incubation at 55°C to inactivate the DNase. RNA was subsequently stored at -80°C and thawed on ice immediately before library preparation.

#### 2.1.4 Library preparation and sequencing

Total RNA sequencing libraries were prepared starting from 8 µL of RNA eluate (plasma) or 40 ng total RNA (FFPE) using the SMARTer Stranded Total RNA-Seq Kit v3 – Pico Input Mammalian (Takara, 634487) according to the manufacturer’s protocol. Equimolar library pools were prepared based on qPCR quantification with KAPA Library Quantification Kit (Roche Diagnostics, Belgium, KK4854). The libraries were paired-end sequenced (2x100 nucleotides) on a NovaSeq 6000 instrument using a NovaSeq S2 kit (Illumina, 20028315) with standard workflow loading of 0.65 nM (2% PhiX). BCL files generated by the Illumina sequencing system were processed using the Illumina Bcl2fastq (v. 2.20) software to generate and demultiplex fastq files. Raw reads were assessed for quality using FastQC (v. 0.11.9) (15). The unique molecular identifier (UMI) sequences in the Pico v3 SMART UMI adapters were first extracted and added to the read name with UMI-tools (v. 1.0.0) (16). Next, the sequencing data were processed with an in-house developed and validated pipeline using cutadapt (v. 1.16) (17), SAMtools (v. 1.9) (18), and STAR (v. 2.7.3a) (19) to obtain deduplicated, high-quality and aligned RNA counts against the human genome (hg38). Ribosomal contamination was assessed using the BBMap tool (v. 38.87) (20). Duplicate reads were identified using the Picard tool (v. 2.21.6) (21). Additional QC statistics were generated by using multiQC (v. 1.9) software package (22). Gene counts were determined with HTSeq (v. 0.11.0) in reverse stranded mode, only considering uniquely mapping reads (23). Ensembl release 91 was used to annotate reads within human genes (24). Circtools (v. 1.2.0) was used to identify, annotate, and quantify back-spliced junction (BSJ) reads from circRNAs (25).

### 2.2 Data analysis

#### 2.2.1 RNA concentration

Blood plasma RNA concentration was determined as previously described (26). Briefly, the mass of the top abundant spike-in control (ERCC00130) was calculated based on the input concentration and volume of spike-in mix added to the sample. The corresponding RNA concentration was then estimated by multiplying the ERCC00130 spike mass by the ratio of total reads mapped to the endogenous human genome and the number of reads mapped to the specific ERCC00130 spike, and finally dividing the obtained mass by the plasma volume of the sample.

#### 2.2.2 Differential abundance analysis

Differential abundance analysis on normalized counts was performed using DESeq2 (v. 1.36.0) (27). Counts were pre-filtered by requiring a minimum of 10 counts in at least half of the samples in one of the compared groups. CircRNA counts were pre-filtered by requiring a minimum of 4 back-splice junction counts in at least half of the samples in one of the compared groups. In the DESeq2 result table, genes with a Benjamini-Hochberg corrected p-value (q-value) below 0.05 were considered differentially abundant. Volcano plots were visualized using EnhancedVolcano (v. 1.14.0) and Venn diagrams were made using VennDiagramm (v. 1.7.3) (28, 29). Concerning the longitudinal differential abundance analysis, the ImpulseDE2 algorithm (v. 1.6.1) was applied on all patients for which all plasma samples during first-line therapy (diagnosis, interim evaluation, and final evaluation) were available using a q-value below 0.05. ImpulseDE2 models the gene-wise abundance trajectories over time with a descriptive single-pulse function, which is based on a negative binomial noise model with dispersion trend smoothing by DESeq2 (30).

#### 2.2.3 COO classification using GEP and IHC

To investigate the performance of a tissue-derived GEP to correctly classify FFPE and plasma samples based on COO as predicted by IHC, both a normalized rank and a standardized abundance procedure were applied (for more details, cfr. **Supplementary Table 1**). The tissue-derived GEP was constructed starting from the 100 best COO class-predicting genes as initially identified in the Lymphochip microarray data by Alizadeh et al., of which 59 genes were retained following the removal of clone duplicates and uncertain clones (3). For the diagnostic plasma samples, besides the tissue-derived GEP, the same procedures were performed using a plasma-derived GEP as input, i.e. the differential abundant genes (DAG) identified between non-GCB and GCB samples in our cohort.

#### 2.2.4 Survival analysis

To determine which genes best correlated with the different prognostic outcomes studied, a univariate Cox model was run for each gene, and only those genes with a LogRank p-value <=0.01 and absolute beta coefficient >=2 were retained. The significant genes were then categorized as favorable and unfavorable genes according to the beta coefficients (a negative beta coefficient represented a hazard ratio <1, meaning that a gene correlated with a favorable outcome; and vice versa for the positive beta coefficients). Subsequently, a hierarchical clustering was performed on the selected genes using a Euclidean distance and average agglomeration method (“stats” R library v. 3.6.2). Next, the clusters were detected using the Dynamic Tree Cut (v. 1.63-1) R library to avoid the arbitrary choice of the dissimilarity cut-off between clusters when performing a fixed height tree cut (31). For each of the clusters, a gene signature was calculated, which was computed as the average expression value of the genes present in the corresponding cluster. The gene signatures together with NCCN-IPI score were used as covariates of a multivariable Cox regression model. Finally, the samples of each prognostic subgroup were divided into a high-risk group and low-risk group according to the gene signatures. High abundance of the unfavorable gene signature defined a high-risk group, while a low abundance defined a low-risk subgroup and vice versa for favorable signatures. Optimal cutoff point analyses were performed with maximally selected rank statistics using the *maxstat* package (v. 0.7-25) (32). Progression-free survival (PFS) was evaluated from the date of enrollment to the date of disease progression, relapse, death from any cause, or date of last follow-up in case of no event. Overall survival (OS) was evaluated from the date of enrollment to the date of death from any cause or date of last follow-up in case of no event. LogRank tests were used to assess differences in the OS and PFS rates calculated by Kaplan–Meier estimates.

#### 2.2.5 Gene set enrichment analysis

A pre-ranked gene set enrichment analysis using GSEA (v. 4.2.3) was performed to explore the functionally enriched pathways and hallmark gene sets related to subgroups, based on the log_2_ transformed fold changes between the different groups obtained from DESeq2 differential abundance analysis (33). Significant enrichment was defined by a false discovery rate ≤ 0.05. Hallmark and Canonical Pathways gene sets were obtained via the Molecular Signatures Database MSigDB (v. 7.5.1) (34). Pathways were up- or downregulated according to the enrichment score (ES) which represents the degree to which a set was overrepresented at the top or bottom of the ranked list.

#### 2.2.6 Statistical analysis

All analyses were conducted using the R statistical software package (v. 4.0.5) (35). Kruskal-Wallis tests were used to compare multiple groups, followed by Wilcoxon rank-sum tests for pairwise comparisons with Bonferroni-Holm multiple testing correction. Significance was defined as q-values smaller than 0.05. Using GeneOverlap (v. 1.32.0), significance of overlap between gene sets was determined by Fisher’s exact test with the odds ratio representing the strength of the association. The universe consisted of all genes obtained in the corresponding differential abundance analysis. A Jaccard index was calculated to assess similarity between the gene sets, with 0 indicating no similarity and 1 indicating identical sets (36). Correlation analysis was carried out using the Pearson correlation coefficient (R) between different metrics.

## 3 Results

### 3.1 Clinical characteristics

A total of 41 DLBCL and 14 PMBCL patients were included. The mean age at inclusion was 65.3 and 37.0 years, respectively (**Table 1**). Most patients had stage IV disease at time of diagnosis, as defined by the Ann Arbor criteria (61% and 57.1%, respectively). Bulky disease was more common in PMBCL compared to DLBCL patients (50% and 22%, respectively). The National Comprehensive Cancer Network International Prognostic Index score (NCCN-IPI) score was low, low-intermediate, high-intermediate, and high in 2.4%, 39.0%, 39.0%, and 19.5% and in 21.4%, 71.4%, 7.1% and 0% of DLBCL and PMBCL patients, respectively (37). Based on the Hans algorithm, 20/41 and 21/41 DLBCL patients were classified as GCB and non-GCB, respectively (4). Double expression for MYC and BCL2 was demonstrated in 17/41 patients. MYC and BCL2 rearrangements were present in 3/41 and 8/41 of DLBCL cases, respectively, without the presence of a DHL. The mean serum lactate dehydrogenase (LDH) value (reference interval 105-250 IU/L) was 428.2 IU/L and 424.6 IU/L and the mean ß2-microglobulin (reference interval 1.09-2.53 mg/l) was 2.4 mg/l and 1.9 mg/l for the DLBCL and PMBCL patients, respectively.

**Table 1:** Demographics, clinical characteristics, first-line treatments, and outcomes of DLBCL patients, PMBCL patients, and healthy controls included in this study.

	DLBCL NOS	PMBCL	healthy controls
clinical characteristics			
sex (m/v)			
*male*	17/41 (41.5%)	8/14 (57.1%)	13/22 (59%)
*female*	24/41 (58.5%)	6/14 (42.9%)	9/22 (41%)
age at diagnosis (yr)	65.41 (+/- 14.94)	37.00 (+/- 14.35)	58.2 (+/- 20.61)
Ann Arbor stage			
*I*	4/41 (9.8%)	1/14 (7.14%)	
*II*	7/41 (17.1%)	4/14 (28.57%)	
*III*	5/41 (12.2%)	1/14 (7.14%)	
*IV*	25/41 (61.0%)	8/14 (57.14%)	
bulky disease	9/41 (22.0%)	7/14 (50.0%)	
NCCN-IPI score			
*low* (0–1)	1/41 (2.4%)	3/14 (21.4%)	
*low-intermediate* (2–3)	16/41 (39.0%)	10/14 (71.4%)	
*high-intermediate* (4–5)	16/41 (39.0%)	1/14 (7.1%)	
*high (>=6)*	8/41 (19.5%)	0/14 (0%)	
tumor characteristics			
*COO: GCB*	20/41 (48.8%)	NA	
*COO: non-GCB*	21/41 (51.2%)	NA	
*DEL*	17/41 (41.5%)	NA	
*MYC expression*	23/41 (56.1%)	3/14 (21.4%)	
*BCL2 expression*	28/41 (68.3%)	6/14 (42.9%)	
*MYC rearrangement*	3/41 (7.3%)	NA	
*BCL2 rearrangement*	8/41 (19.5%)	NA	
first-line treatment			
R-(mini)CHOP	41/41 (100%)	0/14 (0%)	
DA-EPOCH-R	NA	3/14 (21.4%)	
R-ACVBP	NA	11/14 (78.6%)	
intrathecal chemotherapy	23/41 (56.1%)	14/14 (100%)	
laboratory values			
mean LDH (IU/L)	428.15 (+/- 406.25)	424.57 (+/- 258.50)	
mean ß2 microglobulin (mg/l)	2.44 (+/- 0.95)	1.92 (+/- 0.42)	
mean igG (g/l)	9.66 (+/- 5.43)	11.53 (+/- 2.68)	
outcome after first-line therapy			
CR	21/41 (51.2%)	12/14 (85.7%)	
R/R	19/41 (46.3%)	2/14 (14.3%)	
death	1/41 (2.4%)	0/14 (0%)	
outcome of R/R patients after second- and third-line treatments			
CR	8/19 (42.1%)	2/2 (100%)	
death	11/19 (57.9%)	0/2 (0%)	
outcome after any therapy			
CR	29/41 (70.73%)	14/14 (100%)	
death	12/41 (29.27%)	0/14 (0%)	

Percentages and standard deviations are written in parentheses. COO, cell-of-origin; CR, complete remission; DEL, double-expressor lymphoma; DLBCL NOS, diffuse large B-cell lymphoma not otherwise specified; GCB, germinal center B-cell type; LDH, serum lactate dehydrogenase; NCCN-IPI, National Comprehensive Cancer Network International Prognostic Index score; PMBCL, primary mediastinal B-cell lymphoma; R/R, refractory/relapsed disease.

The DLBCL patients were treated with first-line R-(mini)CHOP and the PMBCL patients with dose-intensive rituximab, doxorubicin, cyclophosphamide, vindesine, bleomycin, and prednisone (R-ACVBP) with subsequent consolidation or dose-adjusted EPOCH-rituximab therapy (DA-EPOCH-R) (38, 39). After first-line therapy, 21/41 of DLBCL patients obtained a sustained complete remission (CR), 19/41 had R/R disease, and 1/41 died during first-line treatment. With second- and third-line treatments, CR could be obtained in 8/19 of R/R DLBCL patients. All treatments combined, 29/41 DLBCL patients obtained a CR and 12/41 died. In the PMBCL patients, CR could be obtained in 12/14 of patients after first-line treatment and in 2/2 of R/R patients with second-line treatments. Lastly, a total of 22 healthy age and gender matched controls were included.

### 3.2 cfRNA concentration in blood plasma

**Figure 1:**
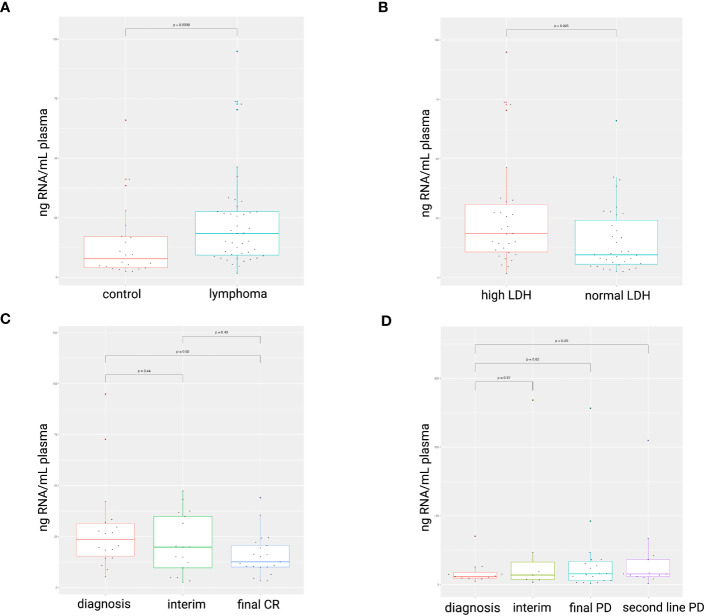
cfRNA concentration (ng RNA/mL plasma) in diagnostic plasma samples. Higher cfRNA concentrations were found in lymphoma patients compared to healthy controls **(A)**. Total cfRNA concentrations were increased in plasma samples with elevated LDH levels compared to normal levels (105-250 IU/L) **(B)**. A decrease in cfRNA concentration was observed in DLBCL patients successfully treated with R-CHOP **(C)**, whereas concentrations remained unchanged in non-responders **(D)**. LDH, serum lactate dehydrogenase; CR, complete response; PD, progressive disease.

The blood plasma cfRNA concentration was higher in the lymphoma patients at the time of diagnosis compared to healthy controls (p = 0.0098; **Figure 1A**). A LDH value above normal reference level at diagnosis was associated with increased cfRNA levels (p=0.025; **Figure 1B**). The correlation between cfRNA concentration and LDH levels was, however, weak in the DLBCL samples (r = 0.4; p = 0.029) with low predictive value (AUC of 0.65) and not significant in the PMBCL samples (r = -0.28; p = 0.36) (**Supplementary Figure 2**). Within the DLBCL samples, a significant decrease in cfRNA concentration was demonstrated between the diagnostic and the post-treatment timepoint in first-line therapy responders (p=0.02), as opposed to non-responders (**Figures 1C, D**). The individual cfRNA evolution of several patients during therapy is visualized in **Supplementary Figure 3**.

### 3.3 cfRNA biomarkers in DLBCL and PMBCL patients

#### 3.3.1 Diagnostic markers

A large number of plasma-derived DAG were identified between DLBCL patients, PMBCL patients and healthy controls in the diagnostic samples. A total of 604, 4076, and 2925 genes were differentially abundant between DLBCL and healthy control samples, between PMBCL and healthy control samples, and between PMBCL and DLBCL samples, respectively. The majority of DAG were protein-coding, but other RNA biotypes included lncRNAs, pseudogenes, snRNAs, snoRNAs, mt-tRNAs, rRNAs, and circRNAs (**Supplementary Figures 4**, **5** and **Supplementary Table 2**). Of the differentially abundant circRNAs, 40.2% of their linear counterparts were also significantly differentially abundant. When both the circRNA and the linear counterpart were significantly differentially abundant, the direction corresponded in 93.9% of the cases.

GSEA between the lymphoma samples and healthy controls showed enrichment of several hallmark gene sets, including interferon type I and II response, interleukin-6/Janus Kinase 2/Signal Transducer and Activator of Transcription 3 (IL-6/JAK/STAT3) signaling, Tumor Necrosis Factor (TNF) alpha signaling via Nuclear factor kappa-light-chain-enhancer of activated B-cells (NF-κB), epithelial mesenchymal transition, inflammatory response, and organization/degradation of the extracellular matrix (ECM). Moreover, compared to PMBCL samples, DLBCL samples showed enrichment for oxidative phosphorylation, KRAS signaling, G2M checkpoint signaling, NOTCH signaling, stabilization of p53, and regulation of Phosphatase and Tensin homolog (PTEN) stability and activity. Compared to the DLBCL samples, the PMBCL samples demonstrated enrichment for coagulation, epithelial mesenchymal transition, MAPK activation, as well as ECM organization and regulation. (**Supplementary Figure 6**).

**Figure 2:**
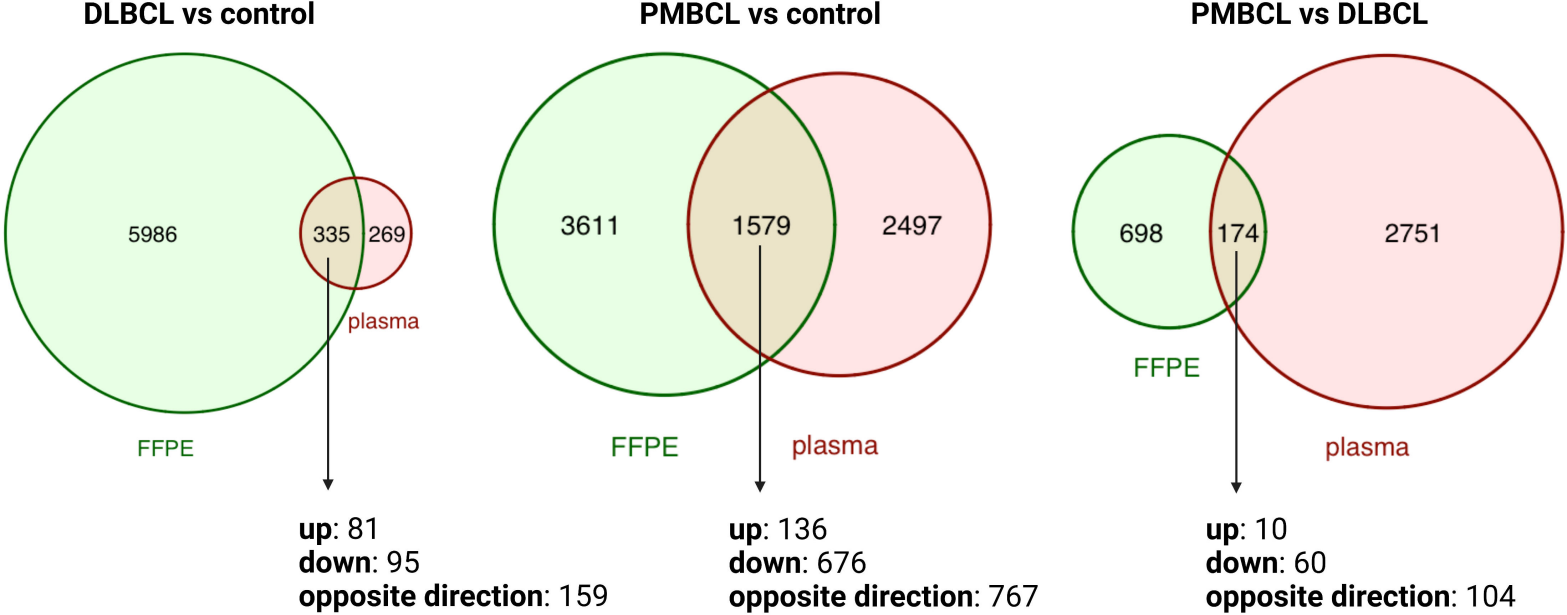
Venn diagrams depicting the overlap between differentially abundant transcripts from matched FFPE and diagnostic plasma samples for DLBCL versus controls, PMBCL versus controls, and PMBCL versus DLBCL. DLBCL, diffuse large B-cell lymphoma; FFPE, formalin-fixed paraffin-embedded tissue; PMBCL, primary mediastinal B-cell lymphoma.

To investigate the differences between the plasma and tissue compartment, the overlap was assessed between the DAG obtained in the diagnostic plasma samples and the matched FFPE samples. (**Figure 2** and **Supplementary Tables 2**, **3**) A total of 335 DAG were shared for DLBCL versus controls (p<0.001; Jaccard Index (JI) 0.051; odds ratio (OR) 3.4), 1579 DAG for PMBCL versus controls (p<0.001; JI 0.21; OR 2.7), and 174 DAG for PMBCL versus DLBCL (p<0.001; JI 0.048; OR 1.8), respectively. This corresponds with 5.30%, 30.42%, and 19.95% of the FFPE DAG also present in the blood plasma, respectively. Of these shared DAG, 52.5%, 51.4%, and 40.2% had the same direction of dysregulation, respectively. The top 10 genes that had the highest Log2FC in FFPE tissue that were also significantly dysregulated in the same direction in the plasma were identified for DLBCL versus controls (upregulated: *LAT2*, *SEMA4A*, *LOX*, *ADAM8*, *PTAFR*; downregulated: *FABP4*, *FAM107A*, *LIFR*, *LPL*, *FMO2*), for PMBCL versus controls (upregulated: *PTGIR*, *CLIP2*, *ANKRD33B*, *ZNF185*, *TREM1*; downregulated: *IGLC2*, *LIFR*, *PDK4*, *PLAC8*, *IGKC*), and for PMBCL versus DLBCL (upregulated: *ANK1*, *PTGIR*, *ANKRD33B*, *SPINT2*, *UNC13B*; downregulated: *PLEKHG1*, *PLAC8*, *FOXP1*, *CCND2*, *PRDX2*).

Secondly, normalized mRNA counts of lymphoma-related genes were compared between DLBCL patients, PMBCL patients, and controls in both the tissue and blood plasma samples, including markers of B-cell differentiation (*CD19*, *CD20*, *PAX5*, *IRF4*, *BCL6*), T-cell differentiation (*CD3*, *CD4*, *CD5*, *CD8*), thymic B-cell differentiation (*CD23*, *MAL*), NF-κB (*REL*, *TRAF1*), cell proliferation (MKI67), activation (*CD30*), and apoptosis (*BCL2*). As expected, many of these genes were differentially abundant between the lymphoma subtypes and healthy controls in tissue samples. These differences were, however, not significant for most markers in the matched plasma samples. Moreover, when comparing DLBCL to control samples, discordant results were seen for *PAX5* (GCB p=0.98, non-GCB p=0.043) and *BCL2* (GCB p=0.61, non-GCB p=0.021) compared to the findings in the FFPE samples. When comparing PMBCL to control samples, concordant results were seen for *BCL2* (p=0.0025) and *CD3* (p=0.0018) and discordant results were noted for *CD20* (p=0.0095), *IRF4* (p=0.0011), and *PAX5* (p=0.041). (**Supplementary Figures 7**, **8**).

Lastly, a differential abundance analysis was performed for cfRNA biomarkers that were previously reported in liquid biopsy studies involving DLBCL and/or PMBCL patients (40–45). For each of these genes, results are shown for both the plasma and FFPE samples (**Table 2**).

**Table 2:** Differential abundance analysis for cfRNA markers that have previously been reported for DLBCL or PMBCL patients.

RNA marker	biotype	sample	DLBCL vs control	non-GCB vs GCB	PMBCL vs control	PMBCL vs DLBCL
**CCND2** (40)	mRNA	FFPE	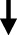 (q=0.026)	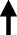 (q=0.0061)	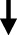 (q<0.001)	NS
		plasma	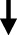 (q=0.003)	NS	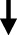 (q<0.001)	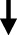 (q=0.0018)
**BCL2** (40)	mRNA	FFPE	NS	NS	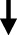 (q<0.001)	NS
		plasma	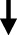 (q=0.038)	NS	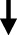 (q=0.0012)	NS
**MYC** (40)	mRNA	FFPE	NS	NS	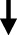 (q=0.0042)	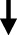 (q=0.0093)
		plasma	NS	NS	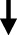 (q=0.002)	NS
**BCL6** (40, 42)	mRNA	FFPE	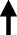 (q<0.001)	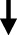 (q=0.033)	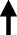 (q<0.001)	NS
		plasma	NS	NS	NS	NS
**FN1** (40)	mRNA	FFPE	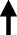 (q<0.001)	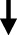 (q=0.02)	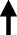 (q<0.001)	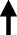 (q=0.012)
		plasma	NS	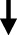 (q=0.037)	NS	NS
**PTEN** (42)	mRNA	FFPE	NS	NS	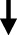 (q=0.0011)	NS
		plasma	NS	NS	NS	NS
**CREBBP** (41)	mRNA	FFPE	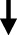 (q<0.001)	NS	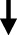 (q<0.001)	NS
		plasma	NS	NS	NS	NS
**LMO2** (40)	mRNA	FFPE	NS	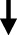 (q=0.033)	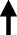 (q<0.001)	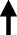 (q<0.001)
		plasma	NS	NS	NS	NS
**TUG1** (43)	lncRNA	FFPE	NS	NS	NS	NS
		plasma	NS	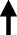 (q=0.013)	NS	NS
**GAS5** (44)	lncRNA	FFPE	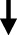 (q<0.001)	NS	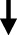 (q=0.0055)	NS
		plasma	NS	NS	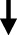 (q=0.0053)	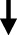 (q=0.033)
**XIST** (44)	lncRNA	FFPE	NS	NS	NS	NS
		plasma	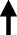 (q=0.011) NS when sex matched	NS	NS	NS
**circAPC** (45)	circRNA	plasma	NS	NS	NS	NS

Genes with q-values >0.05 are labeled as NS. CircRNA, circular RNA; DLBCL, diffuse large B-cell lymphoma; lncRNA, long non-coding RNA; mRNA, messenger RNA; NS, not significant; PMBCL, primary mediastinal B-cell lymphoma. arrow up means upregulated. arrow down means downregulated.

#### 3.3.2 COO and DEL status

**Figure 3:**
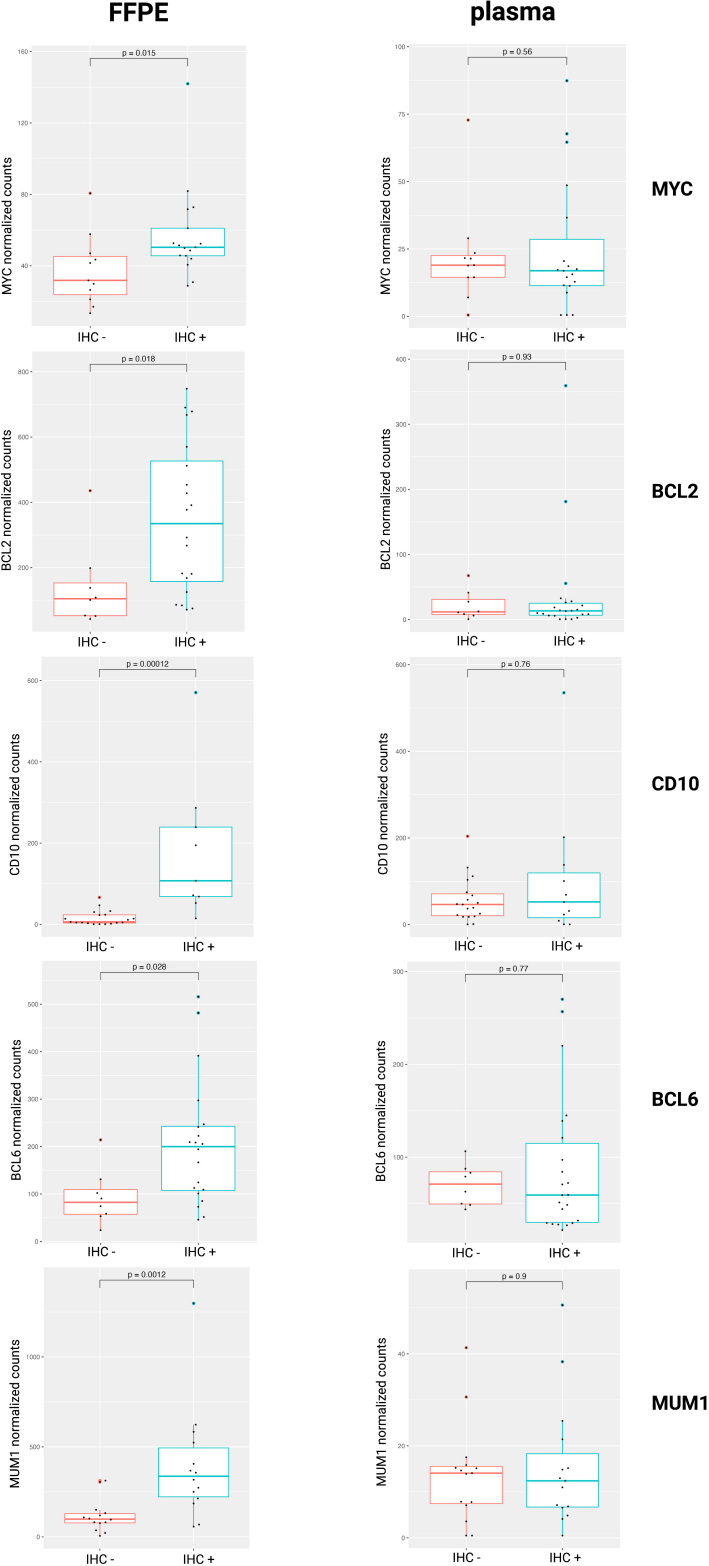
Normalized mRNA counts of *MYC*, *BCL2*, *CD1*0, *BCL6*, and *MUM1* within IHC positive and negative DLBCL patients. DLBCL, diffuse large B-cell lymphoma; FFPE, formalin-fixed paraffin-embedded; IHC, immunohistochemistry; mRNA, messenger RNA.

In clinical practice, IHC markers are used to discern the COO subtypes of DLBCL (e.g., CD10, BCL6, and MUM1 in the Hans algorithm), as well as DEL status (MYC and BCL2) (4, 9). To compare IHC protein expression with the gene abundance profiles in the tissue or blood plasma, normalized mRNA counts for these markers were compared between IHC positive and negative DLBCL patients. Our results showed increased gene counts in the FFPE samples for which there was also positive IHC staining for the corresponding protein. For the plasma samples, however, no significant difference in mRNA counts was observed. (**Figure 3**).

**Figure 4:**
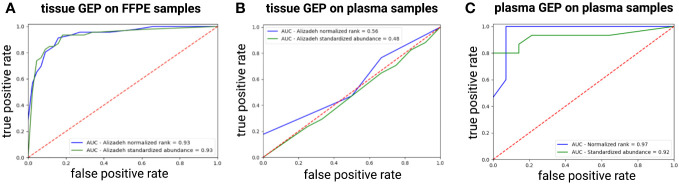
ROC curve showing the performance of the tissue-derived GEP, containing the best COO class-predicting genes as initially identified in the Lymphochip microarray data, to classify DLBCL samples by COO as predicted by the Hans algorithm for FFPE **(A)** and plasma samples **(B)** using both a normalized rank and a standardized abundance approach. Panel **(C)** shows the ROC curve depicting the performance of our plasma-derived GEP to classify DLBCL samples by COO as predicted by the Hans Algorithm using both a normalized rank and a standardized abundance approach **(C)**. AUC, area under the ROC curve; COO, cell-of-origin; DLBCL, diffuse large B-cell lymphoma; FFPE, formalin-fixed paraffin-embedded; GEP, gene expression profiling; ROC, Receiver Operating Characteristic.

Next, we checked if a tissue-derived GEP, containing the best COO class-predicting genes as initially identified in the Lymphochip microarray data by Alizadeh et al., could correctly classify the DLBCL samples (using the Hans algorithm as ground truth) (3). When applied on the FFPE samples, a good concordance with the Hans algorithm could be demonstrated with an accuracy over 85% and an AUC of 0.93 using both a normalized rank and a standardized abundance approach (**Figure 4A**). When applied on the diagnostic plasma samples, however, this tissue-derived classifier failed (accuracy of 0.6 and 0.53 with an AUC 0.56 and 0.48 using a normalized rank and standardized abundance approach, respectively) (**Figure 4B**). Differential abundance analysis on the plasma samples identified a total of 64 DAG between non-GCB and GCB DLBCL cases, as determined by the Hans algorithm (**Supplementary Table 2**). A plasma-derived GEP based on these DAG was able to differentiate GCB from non-GCB patients with high accuracy (accuracy of 92% and 86% with an AUC of 0.97 and 0.92 using a normalized rank and standardized abundance approach, respectively) (**Figure 4C**). Notably, there was no overlap between the tissue- and plasma-derived GEP.

Lastly, no DAG could be identified between DEL and non-DEL samples, illustrating that the transcriptional differences of these subgroups are not clearly reflected in the plasma compartment. This is in line with the observation of only 3 DAG between the matched FFPE samples. (**Supplementary Table 3**).

#### 3.3.3 Prognostic markers in DLBCL

##### 3.3.3.1 Survival analysis

**Figure 5:**
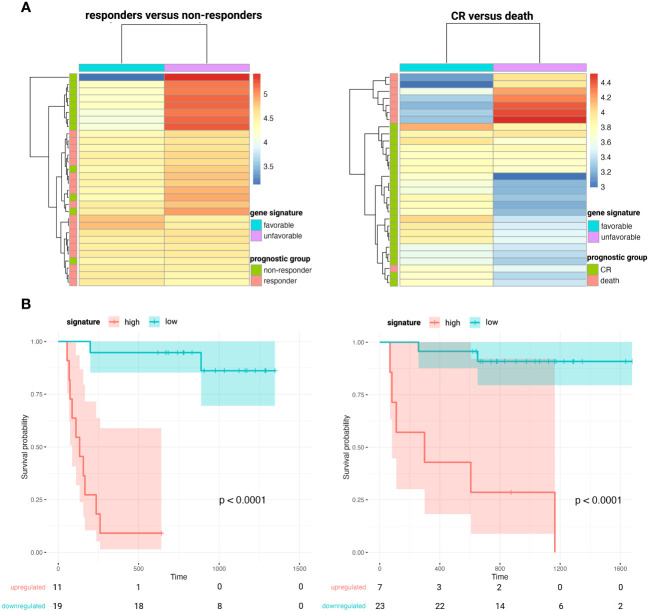
Heatmaps clustering DLBCL responders versus non-responders after first-line R-CHOP therapy (**A**, left) and patients that obtain CR versus death after any therapy (**A**, right) based on the abundance of the corresponding favorable and unfavorable gene signatures. Kaplan-Meier survival curves depicting poorer PFS (**B**, left) and OS (**B**, right) in patients with higher plasma levels of the unfavorable 9- and 5-gene signature, respectively. CR, complete remission; DLBCL, diffuse large B-cell lymphoma; OS, overall survival; PFS, progression-free survival.

When comparing the diagnostic plasma samples of DLBCL responders to non-responders after first line R-CHOP immunochemotherapy, a total of 24 genes were significantly associated with PFS after R-CHOP in univariate cox regression analysis. (**Supplementary Table 4**) The significant genes were clustered according to the beta coefficients (a negative coefficient represented a hazard ratio <1, meaning that a gene correlated with a favorable outcome; and vice versa for a positive beta coefficient). For both the favorable and unfavorable cluster, a gene signature was computed as the average expression value of its genes. A higher abundance of the unfavorable 9-gene signature (*BECN1*, *PRKCB*, *COPA*, *TSC22D3*, *MAP2K3*, *UQCRHL*, *PTMAP4*, *EHD1*, *NAP1L1* pseudogene) was significantly associated with decreased PFS, both in univariate (p<0.001) and in multivariable regression analysis (p<0.001), the latter independent of the NCCN-IPI score. (**Figure 5** and **Supplementary Figure 9**) A similar analysis was performed for the CR versus death groups after any treatment for OS analysis. A total of 10 genes were significantly associated with OS after any therapy in univariate cox regression analysis, of which five were categorized as unfavorable and five as favorable. (**Supplementary Table 4**) A higher abundance of the unfavorable 5-gene signature *(FTH1P7*, *PTMAP4*, *ATF4*, *FTH1P8*, *ARMC7*) was significantly associated with worse OS in both univariate (p<0.001) and multivariable regression analysis (p=0.004). (**Figure 5** and **Supplementary Figure 9**).

##### 3.3.3.2 Longitudinal gene abundance analysis during first line therapy

When comparing matched pre- and post-therapy samples in DLBCL responder patients after successful R-CHOP therapy, a total of 184 DAG were identified. (**Supplementary Table 4**) Among the downregulated genes with the highest log2FC were B-cell related markers, including *MS4A1 (CD20)*, *PAX5*, *IGHD*, *FCRL1*, *CD79A*, *TNFRSF13C*, *IGHM*, *FCER2*, *CD72*, *CD22*, *CARD11*, *IRF4*, *EBF1*, *BLK*, *BLNK*, *STAP1*, *FAM129C*, *BACH2*, *TCL1A*, POU2AF1 and FOXP1. Four of these DAG (*JAK3*, *LRP1*, *PHYKPL*, and *PLXNB2)* were also upregulated at pre-therapy timepoint compared to control samples, but decreased significantly during therapy below healthy control levels, with the opposite being true for *IGF2*. When analyzing the interim time point during therapy, a clear decrease in the aforementioned B-cell related markers was already noted to similar levels as the post-therapy time point. Additionally, a transient increase in neutrophil and inflammation associated markers, including *CD177*, *MMP8*, *CEACAM8*, *OLFM4*, *BPI*, *MS4A3*, and *MPO*, was demonstrated at the interim timepoint. In contrast, within the DLBCL non-responders, only a transient decrease of several B-cell related markers (*IGHM*, *PAX5*, *CD22*, *POU2AF1*) was noted when comparing the diagnostic samples to the interim samples, no DAG could be identified between the diagnostic and the R/R samples, and no transient increase in inflammation associated markers was noted.

Finally, a longitudinal analysis using impulseDE2 was performed to identify gene abundance trajectories that significantly differ between DLBCL responders and non-responders during therapy course (30). A total of 504 cfRNA transcripts were found to be differentially longitudinally abundant between both groups, including PTMAP4 and NAP1L1 pseudogene, which were also significantly associated with PFS at diagnostic timepoint (**Supplementary Table 4** and **Supplementary Figure 10**). As their abundance trajectory patterns differ between responders and non-responders over the course of R-CHOP, this could indicate that these transcripts could potentially serve as longitudinal prognostic markers beyond the diagnostic timepoint.

## 4 Discussion

Cell-free nucleic acids have increasingly been recognized as valuable precision medicine biomarkers in cancer research, including lymphoproliferative malignancies (10, 46–51). Although the origin of cfRNA remains largely unknown, both human and xenograft studies have illustrated its potential to reflect intra- and intertumoral heterogeneity, as well as functional changes during disease course and treatment, both of the tumor and the non-malignant compartment (11, 52, 53). We present the first study to longitudinally explore the total cell-free transcriptome in a cohort of DLBCL and PMBCL patients.

Higher ctDNA levels have been demonstrated in DLBCL patients compared to healthy controls (54, 55). Concordantly, our results showed a higher cfRNA concentration in the DLBCL and PMBCL samples. A significant decrease was observed after successful R-CHOP treatment, potentially indicating that a decline in cfRNA concentration levels by itself may reflect therapy response. CtDNA levels at diagnosis have been shown to correlate with LDH levels, a surrogate marker of tumor burden that is incorporated in the NCCN-IPI score due to its correlation with prognosis in NHL (37, 47, 50, 56–58). No firm correlation could, however, be demonstrated between cfRNA concentration and LDH levels in this study.

A large number of DAG were identified between DLBCL patients, PMBCL patients, and healthy controls. Considering mRNA, multiple widely used lymphoma markers were well reflected in the FFPE tissue, but poorly in the cell-free RNA compartment. Similarly, the majority of FFPE-derived DAG were not differential in the matched blood plasma, and only approximately half of the shared DAG showed the same direction of dysregulation. This finding is in concordance with a previous study in which extracellular mRNA of *CCND2*, *BCL2*, *MYC*, *LMO2*, and *BCL6* was detected in only 14%, 10%, 10%, 10%, and 5% of DLBCL plasma samples, respectively, but in all of the matched tissue samples (40). Similar discrepancies have been reported for non-coding RNAs, in which no correlation between the EV-derived and the tissue derived miRNA repertoire, or even an opposite miRNA expression profile in serum versus matched tumor tissues was demonstrated (59, 60). Potential reasons include variability in (vesicle-mediated) secretion or passive release of RNA molecules in the bloodstream, differences in the rate and manner of degradation, and the shedding of these markers from other tissues besides the tumor compartment. Our overlap analysis pinpointed multiple differentially abundant genes in the same direction in both compartments, likely representing tumor-specific cfRNA markers.

Several diagnostic cfRNA markers have been reported in DLBCL. In contrast to a previous study, decreased G1/S-specific cyclin-D2 (*CCND2*) and B-cell lymphoma 2 (*BCL2*) plasma levels were shown in DLBCL patients compared to healthy controls (40). More specifically *CCND2* levels were decreased in both GCB and non-GCB samples compared to controls, and *BCL2* levels were only significantly lower in the non-GCB samples compared to controls. In the tissue samples, higher *CCND2* levels were found in the non-GCB compared to the GCB tissue samples, which has been reported and associated with an inferior prognosis within this COO subtype (3, 61). Similar to previous findings, B-cell lymphoma 6 (*BCL6*) and Fibronectin 1 (*FN1*) levels were not differentially abundant in the DLBCL plasma samples as opposed to control samples, although our results revealed higher levels in the FFPE samples (40). Concerning lncRNAs, decreased Growth Arrest Specific 5 (*GAS5*), as well as increased Taurine Up-Regulated 1 (*TUG1*) and increased X-inactive specific transcript (*XIST*) plasma levels have been demonstrated in DLBCL compared to control plasma samples (43, 44). In our cohort, *GAS5* levels were found significantly decreased in DLBCL FPPE tissue, but not in blood plasma. *TUG1* levels were found to be upregulated in the plasma of non-GCB DLBCL, compared to GCB DLBCL and control samples. Our results showed higher plasma levels of *XIST*, located on the long arm of the X chromosome, in the DLBCL patients. When comparing sex-matched samples, however, no significant result could be retained, indicating that the difference is sex-based and not disease-driven in our cohort. Lastly, *circAPC*, derived from the host gene adenomatous polyposis coli (*APC*), has been reported as downregulated in DLBCL patients (45). Although a similar trend was visible, statistical significance was not reached.

The COO classification provided a framework on which our current understanding of DLBCL biology is built, classifying tumors based on distinct patterns of GEP into GCB and ABC subtypes that have significant differences in survival (3, 62). Our results showed a good concordance between IHC staining and corresponding mRNA abundance of *CD10*, *BCL6*, and *MUM1* for the DLBCL FFPE tissue, but not the plasma samples. Moreover, a good performance of a tissue-derived GEP, containing the best class-predicting genes as initially identified in the Lymphochip microarray data, to classify FFPE samples according to Hans algorithm COO was demonstrated, comparable with what was previously reported (4, 63). This tissue classifier, however failed for the diagnostic plasma samples. Besides *miR-21* and *miR-155*, no cfRNA markers have been described for COO classification (11, 64, 65). Therefore, a plasma-derived COO classifier was developed, based on the DAG identified in our cohort, which demonstrated a high concordance with the Hans algorithm. Notably, there was no overlap between our plasma-derived and the tissue-derived GEP. Potential reasons include that the latter is mainly based on mRNA and the former also incorporated the non-coding transcriptome, including lncRNA, mt-RNA and pseudogenes, as well as the use of distinct RNA characterization methods. The lack of overlap supports the observation that cfRNA abundance does not necessarily reflect the transcriptomic differences found in the tissue but has a unique abundance profile that besides tumor-derived transcripts also contains healthy tissue-derived signals and exhibits its own dynamics.

Pretreatment ctDNA levels have been shown as independent prognostic marker for event-free survival (EFS), PFS, and OS in DLBCL patients (46, 51, 54, 58, 66). Here, we have established a 9-gene and a 5-gene cfRNA signature, whose increased abundance was negatively associated with PFS and OS, respectively, independent of the NCCN-IPI score. The majority of the protein-coding genes in these signatures have been attributed an oncogenic role and have been associated with adverse prognostic outcomes (67–76). *FTH1P7/8*, *PTMAP4*, and *NAP1L1* pseudogene are pseudogenes of Ferritin Heavy Chain 1 (*FTH1*), Prothymosin Alpha (*PTMA*), and Nucleosome Assembly Protein 1 Like 1 (*NAP1L1*), respectively, which have been found dysregulated in various malignancies (77–79). Pseudogenes are being increasingly recognized as regulators of essential biological processes as they can compete with their parental gene for binding to miRNAs, while others generate small interference RNAs to dampen gene expression or encode functional mutated proteins (78). Protein Kinase C Beta *(PRKCB)* is an integral component of signaling via the B-cell receptor in DLBCL, activating NF-κB and VEGF–mediated angiogenesis, and its tissue expression was shown to be an independent predictor of poor OS (68, 69). Decreased Activating Transcription Factor 4 (*ATF4)* levels have been associated with improved survival probability in DLBCL patients (70). A recent study identified a Sirtuin 3 (SIRT3)–ATF4 axis required to maintain survival of DLBCL cells, regardless of subtype, by enabling them to optimize amino acid uptake and utilization. Targeting *ATF4* translation may, therefore, potentiate the cytotoxic effect of SIRT3 inhibition and serve as a potential therapeutic target (80).

PET-guided treatment of DLBCL remains debatable as direct evidence of improved patient outcomes is still lacking. Outside of a clinical trial, biopsy confirmation of an abnormal interim PET-CT scan is still recommended before switching therapy (81–83). In recent years, serial cell-free nucleic acids analyses have demonstrated potential to complement the predictive value of imaging results, as ctDNA monitoring has shown to facilitate (minimal) residual disease assessment and early relapse detection (46, 47, 56, 58, 66, 84–86). Only few studies have, however, investigated longitudinal abundance of (EV-derived) cfRNA markers (87, 88). When comparing pre- to interim-, and to post-therapy samples in DLBCL responders, a clear decrease in cell-free mRNA of B-cell related markers was noted, many of which have been proposed as potential diagnostic and/or prognostic biomarkers in DLBCL tissue (89–98). Moreover, the interim timepoint showed a clear, transient increase in neutrophil- and inflammation-related markers, reflecting an acute-phase reaction during therapy. In the R/R patients, only a transient decrease at interim timepoint of several B-cell markers was noted, and no acute phase response was discerned, potentially reflecting the poorer response to R-CHOP, and illustrating that repetitive sampling may allow to discern dynamic transcriptomic changes in relation to therapy response.

Our study has several limitations. Our findings have been compared with previous studies that have mainly used RT-qPCR or microarray-based abundance analyses and have collected and processed samples using other blood collection tubes and protocols, which may hamper direct comparison of results. Moreover, specific genes can be differentially enriched in EVs compared to the circulating-free compartment, underlining that caution should be applied when comparing the results of studies investigating different biofluid fractions (11, 88). The impact of these pre-analytical variables on cfRNA abundance results has been increasingly reported (99, 100). Secondly, because of the total RNA sequencing library preparation method, the miRNA fraction is underrepresented in our analyses as compared to the protein-coding and other non-coding RNA classes. Third, although the control samples were relatively equally distributed by sex and age, other variables may influence the cfRNA profile, including comorbidities, lifestyle conditions and medication intake, which may affect differential abundance analysis. Lastly, the gene signatures identified in this study need validation in an independent cohort. Currently, no other dataset with plasma-derived (long) RNA-seq data from a cohort of DLBCL and/or PMBCL patients is publicly available. A cohort is being prospectively collected for validation purposes of the results obtained in this study.

In conclusion, we present the first study to longitudinally explore the total cell-free transcriptome in DLBCL and PMBCL patients using blood plasma samples. A large number of differentially abundant genes were identified and compared against previous cfRNA biomarker studies. Overall, overlap analyses with matched FFPE samples showed that blood plasma has a unique transcriptomic profile, that significantly differs from that of the tissue. Our results demonstrated a good concordance between tissue-derived gene expression and the Hans algorithm for COO classification in FFPE samples, but not in the plasma samples. Therefore, a cfRNA classifier was developed that can accurately determine COO in plasma. Lastly, high plasma levels of a 9-gene signature and a 5-gene signature were associated with inferior PFS and OS in DLBCL patients, respectively, independent of the NCCN-IPI score. Our results may serve as a reference point for future cfRNA studies, and the biomarkers identified may represent potential targets for in-depth functional analysis.

## Data availability statement

The datasets presented in this study can be found in online repositories. The names of the repository/repositories and accession number(s) can be found below: https://ega-archive.org, ID EGAS0000100758 (dataset EGAD00001011679).

## Ethics statement

The studies involving humans were approved by medical Ethical Committee of Ghent University Hospital, Ghent, Belgium (EC/2016/0307). The studies were conducted in accordance with the local legislation and institutional requirements. The participants provided their written informed consent to participate in this study.

## Author contributions

PD, FO, and JV performed study concept and design; PD, EG, JV, and FO performed writing, review, and revision of the paper; PD, EG, JV, and FO provided acquisition, analysis and interpretation of data. EG and PD performed statistical analysis; JVD, DD, JA, KV, MVdL, and KDG provided technical and material support. All authors contributed to the article and approved the submitted version.

## Supplementary material

Supplementary Figure 1 Overview of the blood plasma samples included per time point in the study (total of 168 samples). Response at each timepoint was assessed by PET-CT. CR: complete remission; PD: progressive disease; PET-CT: positron emission tomography/computerized tomography.

Supplementary Figure 2 Correlation between LDH (IU/L) and cfRNA concentration (ng/ml blood plasma) in diagnostic plasma samples of DLBCL (red) and PMBCL (green) samples **(A)**. ROC curve of cfRNA concentration for predicting abnormal LDH levels in diagnostic plasma samples of DLBCL patients (AUC=0.65) **(B)**. AUC: area under the ROC curve; DLBCL: diffuse large B-cell lymphoma; LDH: lactate dehydrogenase; PMBCL: primary mediastinal B-cell lymphoma; ROC: Receiver Operating Characteristic.

Supplementary Figure 3 Evolution of cfRNA concentrations in two responders to first-line treatment **(A)** and two non-responders to first-line treatment **(B)**. CR: complete remission; DLBCL: diffuse large B-cell lymphoma; PD: progressive disease; PMBCL: primary mediastinal B-cell lymphoma; PR: partial response.

Supplementary Figure 4 Differentially abundant genes in the diagnostic plasma samples between different groups for each RNA subclass. CircRNA: circular RNA; DLBCL: diffuse large B-cell lymphoma; GCB: germinal center B-cell; lncRNA: long non-coding RNA; misc_RNA: miscellaneous RNA; mt-tRNA: mitochondrial tRNA; PMBCL: primary mediastinal B-cell lymphoma; rRNA: ribosomal RNA; snRNA: small nuclear RNA; snoRNA: small nucleolar RNA; TEC: To be Experimentally Confirmed.

Supplementary Figure 5 Volcano plots of the differentially abundant genes for the DLBCL versus control, PMBCL versus control, and PMBCL versus DLBCL comparisons in the diagnostic plasma samples (cutoff q-value =<0.05 and |log2FC|>=1) **(A)**. Venn diagram illustrating the overlap of significantly differentially abundant transcripts between the different comparisons. Separate diagrams are shown for all genes, for mRNAs, lncRNAs, and circRNAs subtypes. **(B)** circRNA: circular RNA; DLBCL: diffuse large B-cell lymphoma; lncRNA: long non-coding RNA; log2FC: log2 fold change; mRNA: messenger RNA; PMBCL: primary mediastinal B-cell lymphoma.

Supplementary Figure 6 Gene set enrichment analysis (GSEA) on the diagnostic plasma samples for DLBCL patients versus healthy controls **(A)**, PMBCL patients versus healthy controls **(B)**, and PBMCL versus DLBCL patients **(C)**. DLBCL: diffuse large B-cell lymphoma; PMBCL: primary mediastinal B-cell lymphoma.

Supplementary Figure 7 Normalized mRNA counts for *CD3*, *CD4*, *CD5*, *CD8*, *CD19*, *CD20*, *CD23*, *CD30*, *PAX5*, *BCL2*, *BCL6*, *MKI67*, *IRF4*, *MAL*, *REL*, and *TRAF1* within the FFPE samples of healthy controls, GCB-DLBCL patients, non-GCB-DLBCL patients, and PMBCL patients. Multiple testing corrected p-values are shown for pairwise comparisons. DLBCL: diffuse large B-cell lymphoma; GCB: germinal center derived B-cell lymphoma; PMBCL: primary mediastinal B-cell lymphoma.

Supplementary Figure 8 Normalized mRNA counts of *CD3*, *CD4*, *CD5*, *CD8*, *CD19*, *CD20*, *CD23*, *CD30*, *PAX5*, *BCL2*, *BCL6*, *MKI67*, *IRF4*, *MAL*, *REL*, and *TRAF1* within the diagnostic plasma samples of healthy controls, GCB-DLBCL patients, non-GCB-DLBCL patients, and PMBCL patients. Multiple testing corrected p-values are shown for pairwise comparisons. DLBCL: diffuse large B-cell lymphoma; GCB: germinal center derived B-cell lymphoma; PMBCL: primary mediastinal B-cell lymphoma.

Supplementary Figure 9 The genes in the unfavorable gene signatures **(A)** that are significantly associated with PFS (left) and OS (right), respectively, in univariate **(B)** and multivariable analysis **(C)**, the latter also including the NCCN-IPI score. CI: confidence interval; HR: hazard ratio; NCCN-IPI: National Comprehensive Cancer Network International Prognostic Index score; OS: overall survival; PFS: progression-free survival.

Supplementary Figure 10 Abundance trajectories of PTMAP4 and NAP1L1 pseudogene between DLBCL responder and non-responder patients (n=27 per timepoint) in matched samples at diagnosis, at interim evaluation and at final evaluation after R-CHOP therapy. Multiple testing corrected p-values are shown for pairwise comparisons. DLBCL: diffuse large B-cell lymphoma; R-CHOP: rituximab, cyclophosphamide, vincristine, doxorubicin, and prednisone.

Supplementary Table 1 Procedure to evaluate the performance of a tissue- and plasma-derived GEP for IHC COO classification.

Supplementary Table 2 Overview of plasma-derived DAGs for DLBCL versus healthy controls, PMBCL versus healthy controls, PMBCL versus DLBCL patients, and GCB versus non-GCB samples. For each comparison, the top 500 DAG are shown according to the absolute log2FC. DAGs: differentially abundant genes; DLBCL: diffuse large B-cell lymphoma; GCB: germinal center B-cell type; log2FC: log2 fold change; PMBCL: primary mediastinal B-cell lymphoma.

Supplementary Table 3 Overview of FFPE-derived DAGs for DLBCL versus healthy controls, PMBCL versus healthy controls, PMBCL versus DLBCL patients, non-GCB versus GCB samples, and non-DEL versus DEL samples. For each comparison, the top 500 DAG are shown according to the absolute log2FC. DAGs: differentially abundant genes; DEL: double expressor lymphoma; DLBCL: diffuse large B-cell lymphoma; log2FC: log2 fold change; GCB: germinal center B-cell type; PMBCL: primary mediastinal B-cell lymphoma.

Supplementary Table 4 Overview of the genes in the diagnostic plasma samples of DLBCL patients that were significantly associated with PFS and OS in univariate cox regression analysis, overview of the plasma-derived DAGs between the diagnostic, interim evaluation, and final evaluation timepoints for DLBCL responders and non-responders, as well as an overview of the DAG identified in the ImpulseDE2 analysis. DAGs: differentially abundant genes; DLBCL: diffuse large B-cell lymphoma; log2FC: log2 fold change.

## Glossary

**Table:**

ABC	activated B-cell lymphoma
APC	adenomatous polyposis coli
ATF4	Decreased Activating Transcription Factor 4
BCL2	B-cell lymphoma 2
BCL6	B-cell lymphoma 6
CCND2	G1/S-specific cyclin-D2
CD10	Membrane Metalloendopeptidase
CREBBP	CREB Binding Protein
cfDNA	cell-free DNA
cfRNA	cell-free RNA
circRNA	circular RNA
COO	cell-of-origin
CR	complete remission
ctDNA	circulating tumor DNA
DA-EPOCH-R	dose-adjusted EPOCH-rituximab therapy
DAG	differentially abundant gene
DEL	double-expressor lymphoma
DFS	disease-free survival
DHL	double-hit lymphoma
DLBCL	diffuse large B-cell lymphoma
ECM	extracellular matrix
EFS	event-free survival
ES	enrichment score
EV	extracellular vesicle
FDG-PET/CT	F-fluorodeoxyglucose Positron Emission Tomography/Computerized Tomography
FFPE	formalin-fixed paraffin-embedded tissue
FISH	Fluorescence *in situ* hybridization
FN1	Fibronectin 1
GAS5	Growth Arrest Specific 5
GEP	gene expression profiling
GSEA	gene set enrichment analysis
IGF	Insulin-like Growth Factor
IHC	immunohistochemistry
IL2	interleukin-2
IL6	interleukin-6
IRF4	Interferon Regulatory Factor 4
JAK	Janus kinase
JI	Jaccard Index
LDH	lactate dehydrogenase
LMO2	LIM domain only 2
lncRNA	long non-coding RNA
Log2FC	log2 fold change
misc_RNA	miscellaneous RNA
mRNA	messenger RNA
mt-tRNA	mitochondrial transfer RNA
MYC	MYC proto-oncogene protein
NCCN-IPI	National Comprehensive Cancer Network International Prognostic Index score
NF-κB	Nuclear factor kappa-light-chain-enhancer of activated B-cells
NHL	non-Hodgkin’s lymphoma
OS	overall survival
PD	progressive disease
PFS	progression-free survival
PMBCL	primary mediastinal B-cell lymphoma
PR	partial response
PTEN	Phosphatase and Tensin homolog
R-ACVBP	dose-intensive rituximab, doxorubicin, cyclophosphamide, vindesine, bleomycin, and prednisone
R-CHOP	rituximab, cyclophosphamide, vincristine, doxorubicin, and prednisone
ROC	Receiver Operating Characteristic
R/R	relapse/refractory disease
rRNA	ribosomal RNA
RT-qPCR	reverse transcription quantitative real-time PCR
SD	stable disease
SIRT3	Sirtuin 3
snRNA	small nuclear RNA
snoRNA	small nucleolar RNA
STAT3	signal transducer and activator of transcription 3
TGF-β	Transforming Growth Factor Beta
TNF	Tumor Necrosis Factor
TUG1	Taurine Up-Regulated 1
UMI	Unique Molecular Identifier
VEGF	vascular endothelial growth factor
WHO	World Health Organization
XIST	X-inactive specific transcript.
