# Supplementary material for: Exploring the cell-free total RNA transcriptome in diffuse large B-cell lymphoma and primary mediastinal B-cell lymphoma patients as biomarker source in blood plasma liquid biopsies
Source: Front Oncol. 2023 Oct 25;13:1221471. doi: 10.3389/fonc.2023.1221471 (PMC10634215; doi:10.3389/fonc.2023.1221471)
Supplement: Supplementary Figure 1 — Overview of the blood plasma samples included per time point in the study (total of 168 samples). Response at each timepoint was assessed by PET-CT. CR: complete remission; PD: progressive disease; PET-CT: positron emission tomography/computerized tomography. [file DataSheet_1.zip › Supplementary_Figure_Table_captions.docx]

**Supplementary Figure captions**

Supplementary Figure 1. Overview of the blood plasma samples included per time point in the study (total of 168 samples). Response at each timepoint was assessed by PET-CT. CR: complete remission; PD: progressive disease; PET-CT: positron emission tomography/computerized tomography.

Supplementary Figure 2. Correlation between LDH (IU/L) and cfRNA concentration (ng/ml blood plasma) in diagnostic plasma samples of DLBCL (red) and PMBCL (green) samples (A). ROC curve of cfRNA concentration for predicting abnormal LDH levels in diagnostic plasma samples of DLBCL patients (AUC=0.65) (B). AUC: area under the ROC curve; DLBCL: diffuse large B-cell lymphoma; LDH: lactate dehydrogenase; PMBCL: primary mediastinal B-cell lymphoma; ROC: Receiver Operating Characteristic.

Supplementary Figure 3. Evolution of cfRNA concentrations in two responders to first-line treatment (A) and two non-responders to first-line treatment (B). CR: complete remission; DLBCL: diffuse large B-cell lymphoma; PD: progressive disease; PMBCL: primary mediastinal B-cell lymphoma; PR: partial response.

Supplementary Figure 4. Differentially abundant genes in the diagnostic plasma samples between different groups for each RNA subclass. CircRNA: circular RNA; DLBCL: diffuse large B-cell lymphoma; GCB: germinal center B-cell; lncRNA: long non-coding RNA; misc_RNA: miscellaneous RNA; mt-tRNA: mitochondrial tRNA; PMBCL: primary mediastinal B-cell lymphoma; rRNA: ribosomal RNA; snRNA: small nuclear RNA; snoRNA: small nucleolar RNA; TEC: To be Experimentally Confirmed.

Supplementary Figure 5. Volcano plots of the differentially abundant genes for the DLBCL versus control, PMBCL versus control, and PMBCL versus DLBCL comparisons in the diagnostic plasma samples (cutoff q-value =<0.05 and |log2FC|>=1) (A). Venn diagram illustrating the overlap of significantly differentially abundant transcripts between the different comparisons. Separate diagrams are shown for all genes, for mRNAs, lncRNAs, and circRNAs subtypes. (B) CircRNA: circular RNA; DLBCL: diffuse large B-cell lymphoma; lncRNA: long non-coding RNA; log2FC: log2 fold change; mRNA: messenger RNA; PMBCL: primary mediastinal B-cell lymphoma.

Supplementary Figure 6. Gene set enrichment analysis (GSEA) on the diagnostic plasma samples for DLBCL patients versus healthy controls (A), PMBCL patients versus healthy controls (B), and PBMCL versus DLBCL patients (C). DLBCL: diffuse large B-cell lymphoma; PMBCL: primary mediastinal B-cell lymphoma.

Supplementary Figure 7. Normalized mRNA counts for *CD3*, *CD4*, *CD5*, *CD8*, *CD19*, *CD20*, *CD23*, *CD30*, *PAX5*, *BCL2*, *BCL6*, *MKI67*, *IRF4*, *MAL*, *REL*, and *TRAF1* within the FFPE samples of healthy controls, GCB-DLBCL patients, non-GCB-DLBCL patients, and PMBCL patients. Multiple testing corrected p-values are shown for pairwise comparisons. DLBCL: diffuse large B-cell lymphoma; GCB: germinal center derived B-cell lymphoma; PMBCL: primary mediastinal B-cell lymphoma.

Supplementary Figure 8. Normalized mRNA counts of *CD3*, *CD4*, *CD5*, *CD8*, *CD19*, *CD20*, *CD23*, *CD30*, *PAX5*, *BCL2*, *BCL6*, *MKI67*, *IRF4*, *MAL*, *REL*, and *TRAF1* within the diagnostic plasma samples of healthy controls, GCB-DLBCL patients, non-GCB-DLBCL patients, and PMBCL patients. Multiple testing corrected p-values are shown for pairwise comparisons. DLBCL: diffuse large B-cell lymphoma; GCB: germinal center derived B-cell lymphoma; PMBCL: primary mediastinal B-cell lymphoma.

Supplementary Figure 9. The genes in the unfavorable gene signatures (A) that are significantly associated with PFS (left) and OS (right), respectively, in univariate (B) and multivariable analysis (C), the latter also including the NCCN-IPI score. CI: confidence interval; HR: hazard ratio, NCCN-IPI: National Comprehensive Cancer Network International Prognostic Index score; OS: overall survival; PFS; progression-free survival.

Supplementary Figure 10. Abundance trajectories of PTMAP4 and NAP1L1 pseudogene between DLBCL responder and non-responder patients (n=27 per timepoint) in matched samples at diagnosis, at interim evaluation and at final evaluation after R-CHOP therapy. Multiple testing corrected p-values are shown for pairwise comparisons. DLBCL: diffuse large B-cell lymphoma; R-CHOP: rituximab, cyclophosphamide, vincristine, doxorubicin, and prednisone.

**Supplementary Table captions**

Supplementary Table 1. Procedure to evaluate the performance of a tissue- and plasma-derived GEP for IHC COO classification.

Supplementary Table 2. Overview of plasma-derived DAGs for DLBCL versus healthy controls, PMBCL versus healthy controls, PMBCL versus DLBCL patients, and GCB versus non-GCB samples. For each comparison, the top 500 DAG are shown according to the absolute log2FC. DAGs: differentially abundant genes; DLBCL: diffuse large B-cell lymphoma; GCB: germinal center B-cell type; log2FC: log2 fold change; PMBCL: primary mediastinal B-cell lymphoma.

Supplementary Table 3. Overview of FFPE-derived DAGs for DLBCL versus healthy controls, PMBCL versus healthy controls, PMBCL versus DLBCL patients, non-GCB versus GCB samples, and non-DEL versus DEL samples. For each comparison, the top 500 DAG are shown according to the absolute log2FC. DAGs: differentially abundant genes; DEL: double expressor lymphoma; DLBCL: diffuse large B-cell lymphoma; log2FC: log2 fold change; GCB: germinal center B-cell type; PMBCL: primary mediastinal B-cell lymphoma.

Supplementary Table 4. Overview of the genes in the diagnostic plasma samples of DLBCL patients that were significantly associated with PFS and OS in univariate cox regression analysis, overview of the plasma-derived DAGs between the diagnostic, interim evaluation, and final evaluation timepoints for DLBCL responders and non-responders, as well as an overview of the DAG identified in the ImpulseDE2 analysis. DAGs: differentially abundant genes; DLBCL: diffuse large B-cell lymphoma; log2FC: log2 fold change.
