# Supplementary material for: Exploring the cell-free total RNA transcriptome in diffuse large B-cell lymphoma and primary mediastinal B-cell lymphoma patients as biomarker source in blood plasma liquid biopsies
Source: Front Oncol. 2023 Oct 25;13:1221471. doi: 10.3389/fonc.2023.1221471 (PMC10634215; doi:10.3389/fonc.2023.1221471)
Supplement: Supplementary Figure 1 — Overview of the blood plasma samples included per time point in the study (total of 168 samples). Response at each timepoint was assessed by PET-CT. CR: complete remission; PD: progressive disease; PET-CT: positron emission tomography/computerized tomography. [file DataSheet_1.zip › Supplementary_Table1.docx]

Supplementary Table 1: procedure to evaluate the performance of a tissue- and plasma-derived GEP for IHC COO classification.

In both the FFPE and plasma dataset, the gene expression matrix was normalized (1A) and the samples were ranked according to the corresponding expression value (1B). At this point, two separate procedures were performed: a normalized rank and a standardized abundance method. In the former method, for each sample, the ranks for each gene across all samples were summed for both GCB and ABC subclasses according to the GEP classification, followed by a scaling of these two sums by dividing them by the product of the total number of samples and the number of genes for that subclass. Finally, the two rank sums were divided by the product of the total number of samples and the number of genes for that class (e.g., 4 samples * 2 ABC Genes = 8) and subtracted in order to obtain the final score (1C). In the latter method, the sample ranks were log transformed and standardized (i.e., mean centered and autoscaled) (1E). Next, the ABC and GCB means log rank were computed for each sample and finally the score was obtained as the difference between the two means (GCB - ABC) (1E). A “-1” value represented a pure ABC classification, while a “+1” value represented a GCB classification. For both methods, different thresholds were applied to classify a sample as GCB or ABC subtype with the optimal threshold being defined as the one that maximized the accuracy between the GEP on the one hand, and the Hans IHC classification on the other hand. In the table example below, a threshold of 0 was used.

| **Table 1A** |  | ABC | GCB | ABC | GCB |
| --- | --- | --- | --- | --- | --- |
|  |  | G1 | G2 | G3 | G4 |
| ABC | S01 | 2.58 | 12.18 | 197.22 | 512.13 |
| GCB | S02 | 1.47 | 30.32 | 43.16 | 842.76 |
| ABC | S03 | 2.08 | 10.52 | 198.94 | 631.99 |
| ABC | S04 | 2.22 | 14.98 | 214.08 | 534.38 |

| **Table 1B** |  | ABC | GCB | ABC | GCB |
| --- | --- | --- | --- | --- | --- |
|  |  | G1 | G2 | G3 | G4 |
| ABC | S01 | 1 | 3 | 3 | 4 |
| GCB | S02 | 4 | 1 | 4 | 1 |
| ABC | S03 | 3 | 4 | 2 | 2 |
| ABC | S04 | 2 | 2 | 1 | 3 |

| **Table 1C** | Rank Sum (ABC) | Rank Sum (GCB) | Rescaled Rank Sum  (ABC) | Rescaled Rank Sum (GCB) | Rescaled Rank Sum Difference (ABC-GCB) | Classification |
| --- | --- | --- | --- | --- | --- | --- |
| S01 | 4 | 7 | 0.5 | 0.88 | -0.38 | ABC |
| S02 | 8 | 2 | 1 | 0.25 | 0.75 | GCB |
| S03 | 5 | 6 | 0.63 | 0.75 | -0.12 | ABC |
| S04 | 3 | 5 | 0.38 | 0.63 | -0.25 | ABC |

| **Table 1D** |  | ABC | GCB | ABC | GCB |
| --- | --- | --- | --- | --- | --- |
|  |  | G1 | G2 | G3 | G4 |
| ABC | S01 | 1 | -0.51 | 0.77 | -0.4 |
| GCB | S02 | -1.4 | 1.42 | -2.49 | 0.66 |
| ABC | S03 | 0.1 | -0.85 | 0.78 | 0.04 |
| ABC | S04 | 0.4 | -0.06 | 0.94 | -0.31 |

| **Table 1E** | Mean (ABC) | Mean (GCB) | Log-Stand Score (GCB-ABC) | Classification |
| --- | --- | --- | --- | --- |
| S01 | 0.89 | -0.46 | -1.35 | ABC |
| S02 | -1.95 | 1.04 | 2.99 | GCB |
| S03 | 0.44 | -0.41 | -0.85 | ABC |
| S04 | 0.67 | -0.19 | -0.86 | ABC |
