# Supplementary figures and images for: Exploring the cell-free total RNA transcriptome in diffuse large B-cell lymphoma and primary mediastinal B-cell lymphoma patients as biomarker source in blood plasma liquid biopsies
Source: Front Oncol. 2023 Oct 25;13:1221471. doi: 10.3389/fonc.2023.1221471 (PMC10634215; doi:10.3389/fonc.2023.1221471)

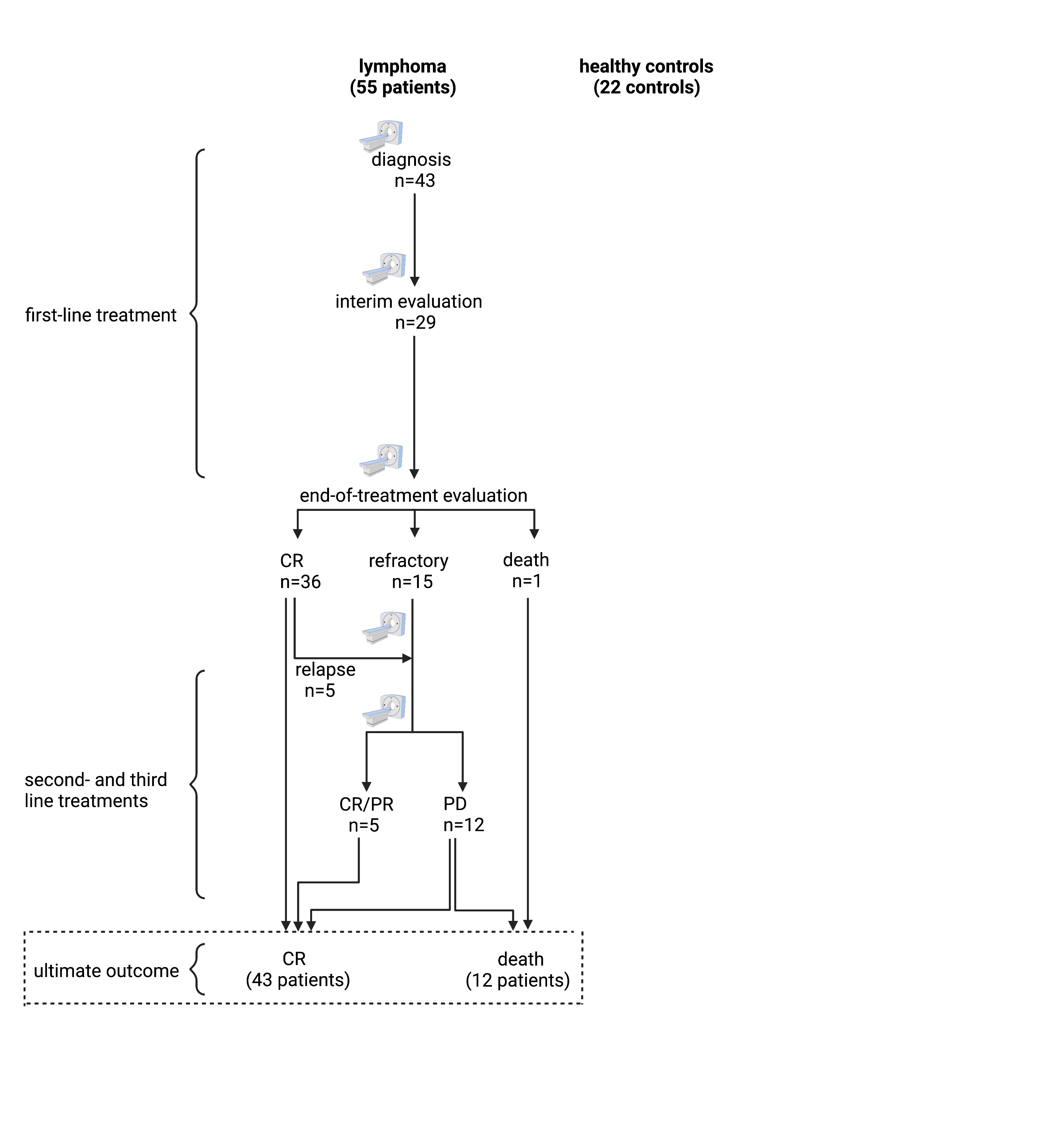

Supplement: Supplementary Figure 1 — Overview of the blood plasma samples included per time point in the study (total of 168 samples). Response at each timepoint was assessed by PET-CT. CR: complete remission; PD: progressive disease; PET-CT: positron emission tomography/computerized tomography. [file DataSheet_1.zip › Supplementary_Figure1.png]

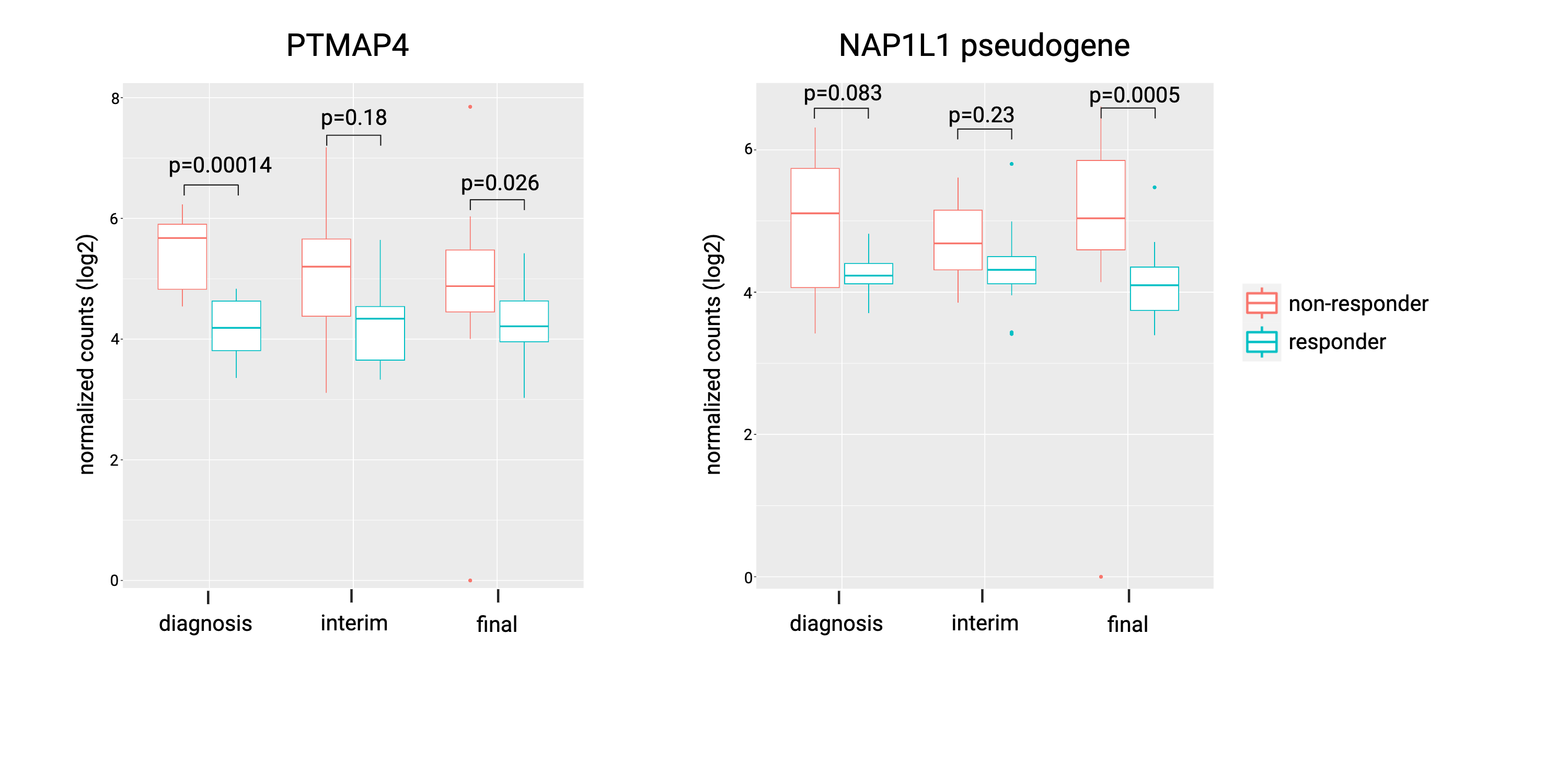

Supplement: Supplementary Figure 1 — Overview of the blood plasma samples included per time point in the study (total of 168 samples). Response at each timepoint was assessed by PET-CT. CR: complete remission; PD: progressive disease; PET-CT: positron emission tomography/computerized tomography. [file DataSheet_1.zip › Supplementary_Figure10.png]

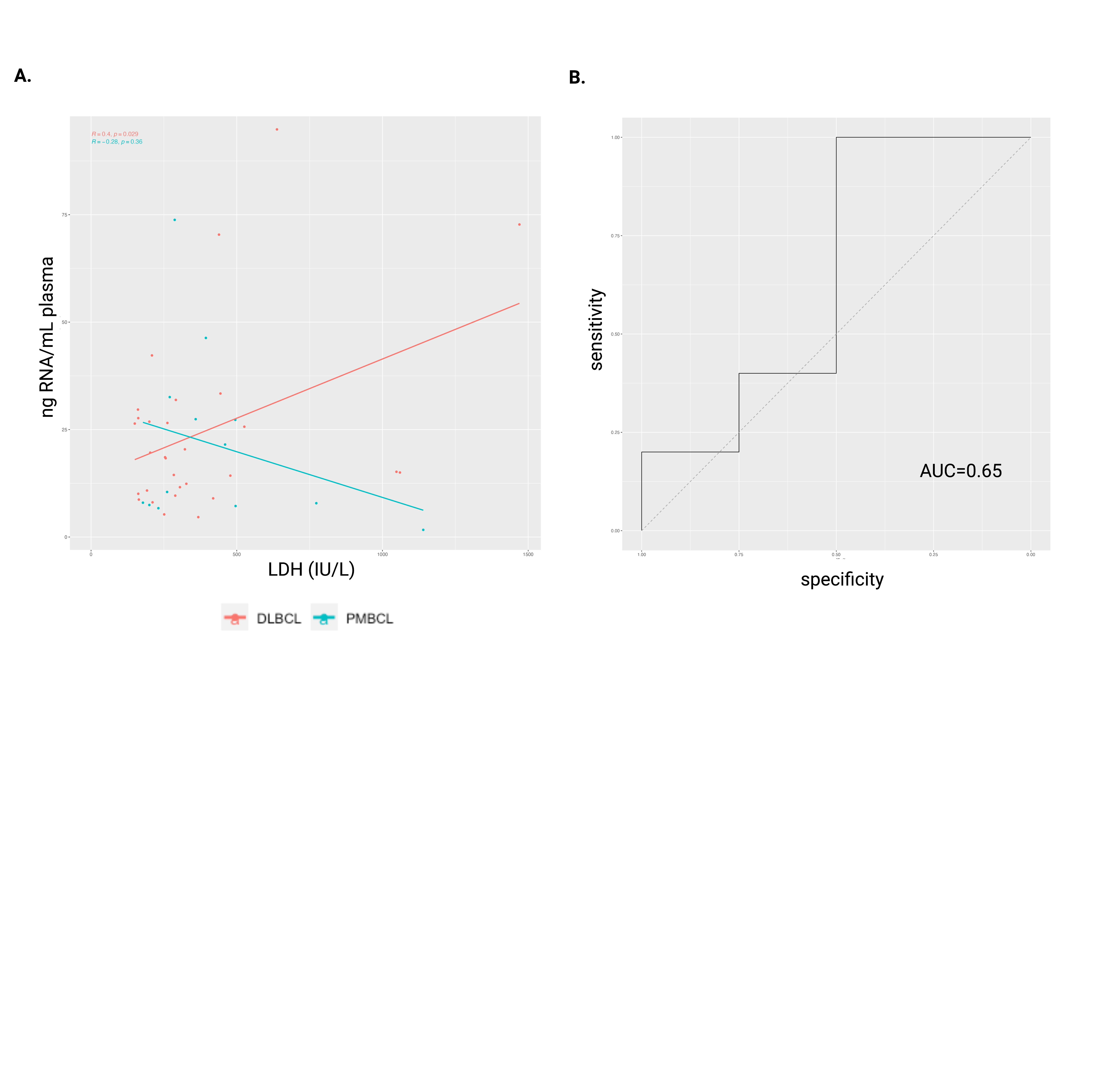

Supplement: Supplementary Figure 1 — Overview of the blood plasma samples included per time point in the study (total of 168 samples). Response at each timepoint was assessed by PET-CT. CR: complete remission; PD: progressive disease; PET-CT: positron emission tomography/computerized tomography. [file DataSheet_1.zip › Supplementary_Figure2.png]

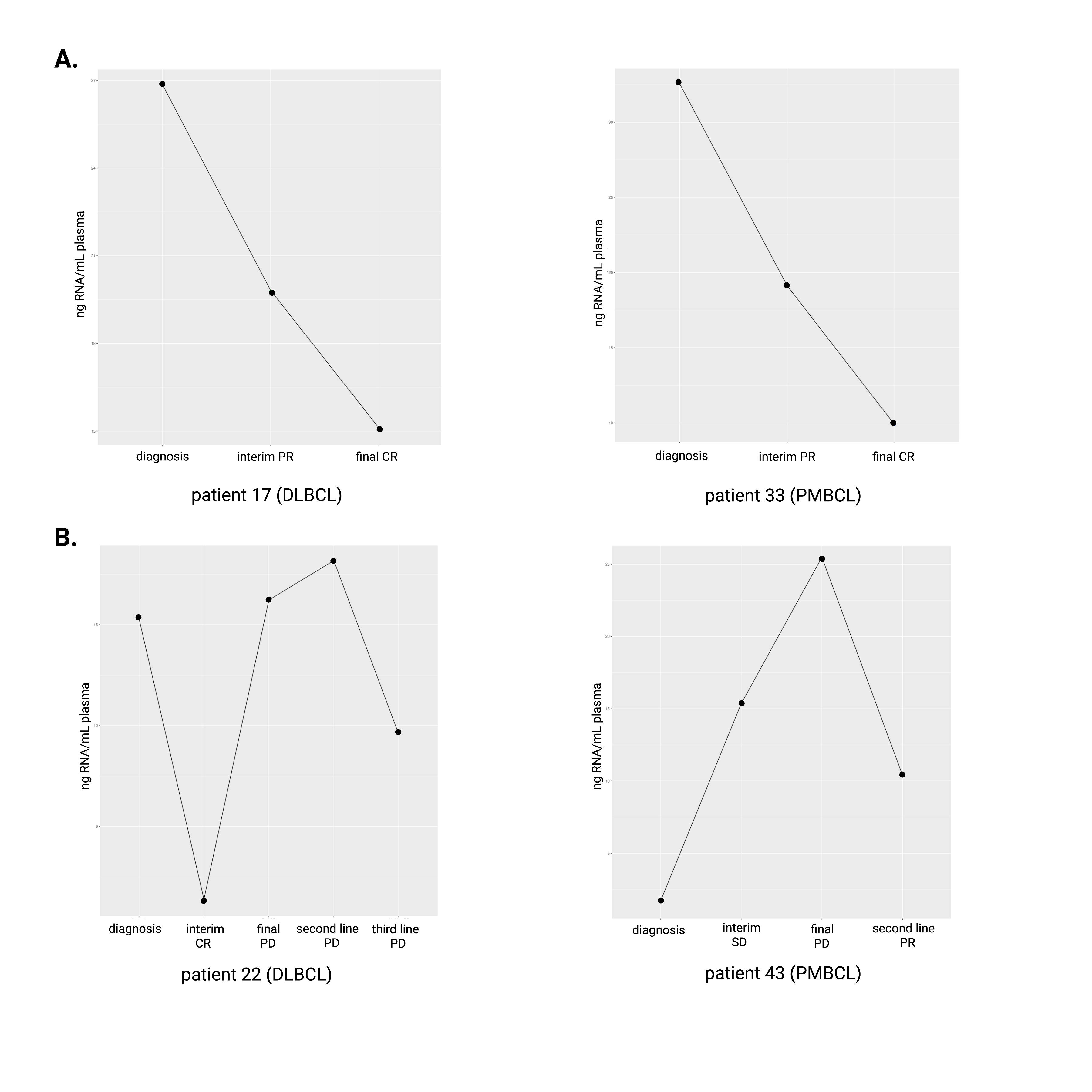

Supplement: Supplementary Figure 1 — Overview of the blood plasma samples included per time point in the study (total of 168 samples). Response at each timepoint was assessed by PET-CT. CR: complete remission; PD: progressive disease; PET-CT: positron emission tomography/computerized tomography. [file DataSheet_1.zip › Supplementary_Figure3.png]

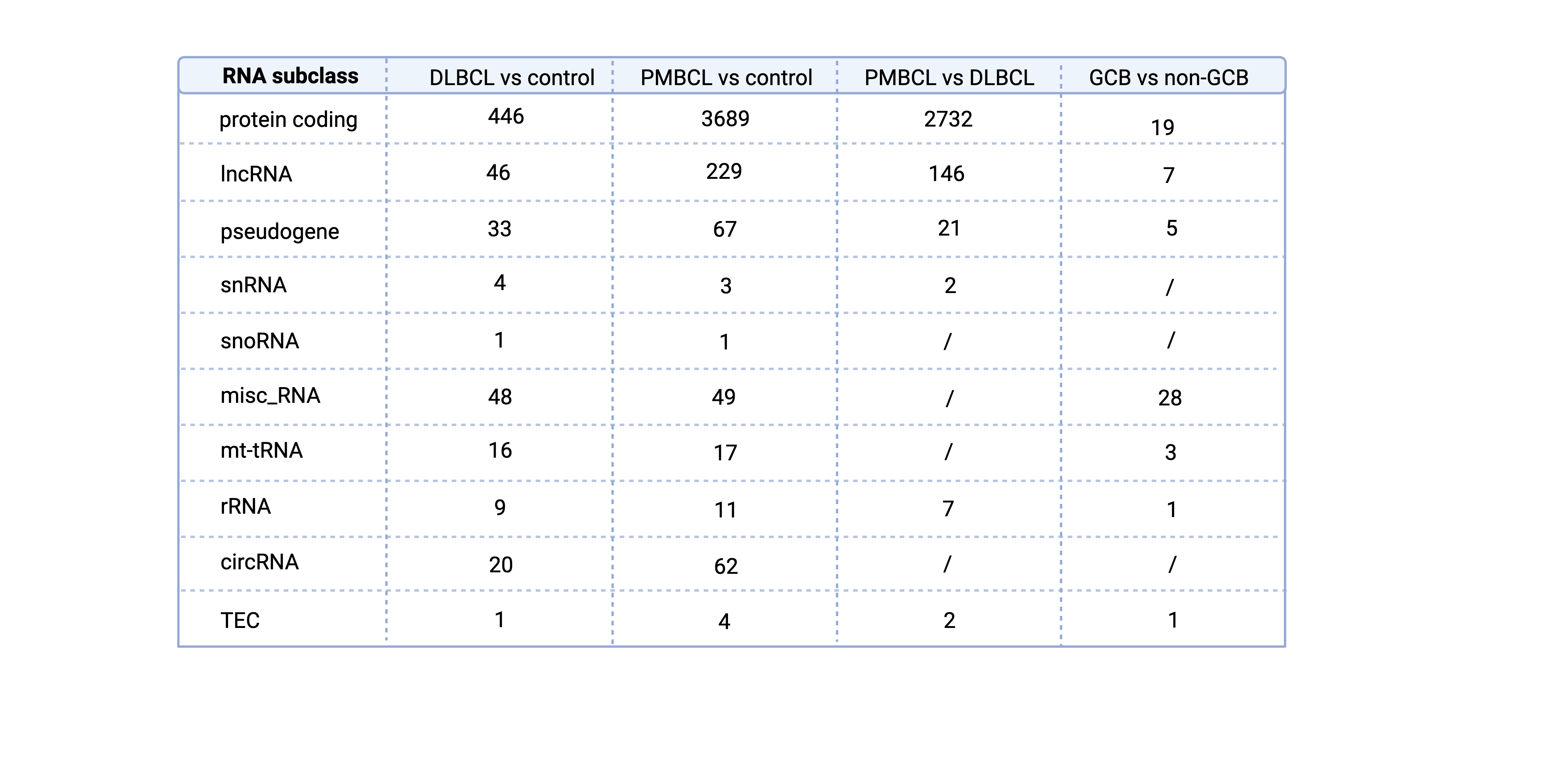

Supplement: Supplementary Figure 1 — Overview of the blood plasma samples included per time point in the study (total of 168 samples). Response at each timepoint was assessed by PET-CT. CR: complete remission; PD: progressive disease; PET-CT: positron emission tomography/computerized tomography. [file DataSheet_1.zip › Supplementary_Figure4.png]

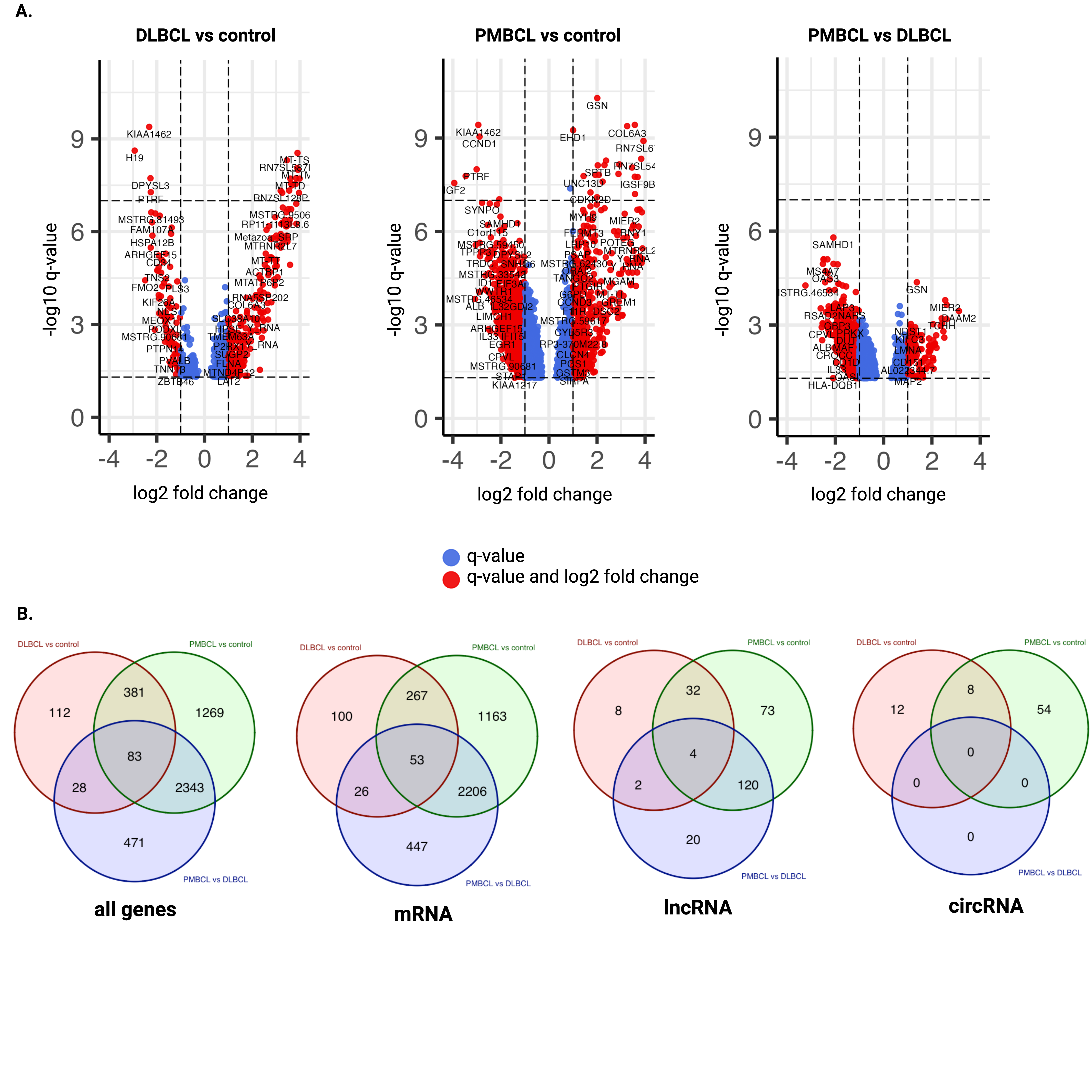

Supplement: Supplementary Figure 1 — Overview of the blood plasma samples included per time point in the study (total of 168 samples). Response at each timepoint was assessed by PET-CT. CR: complete remission; PD: progressive disease; PET-CT: positron emission tomography/computerized tomography. [file DataSheet_1.zip › Supplementary_Figure5.png]

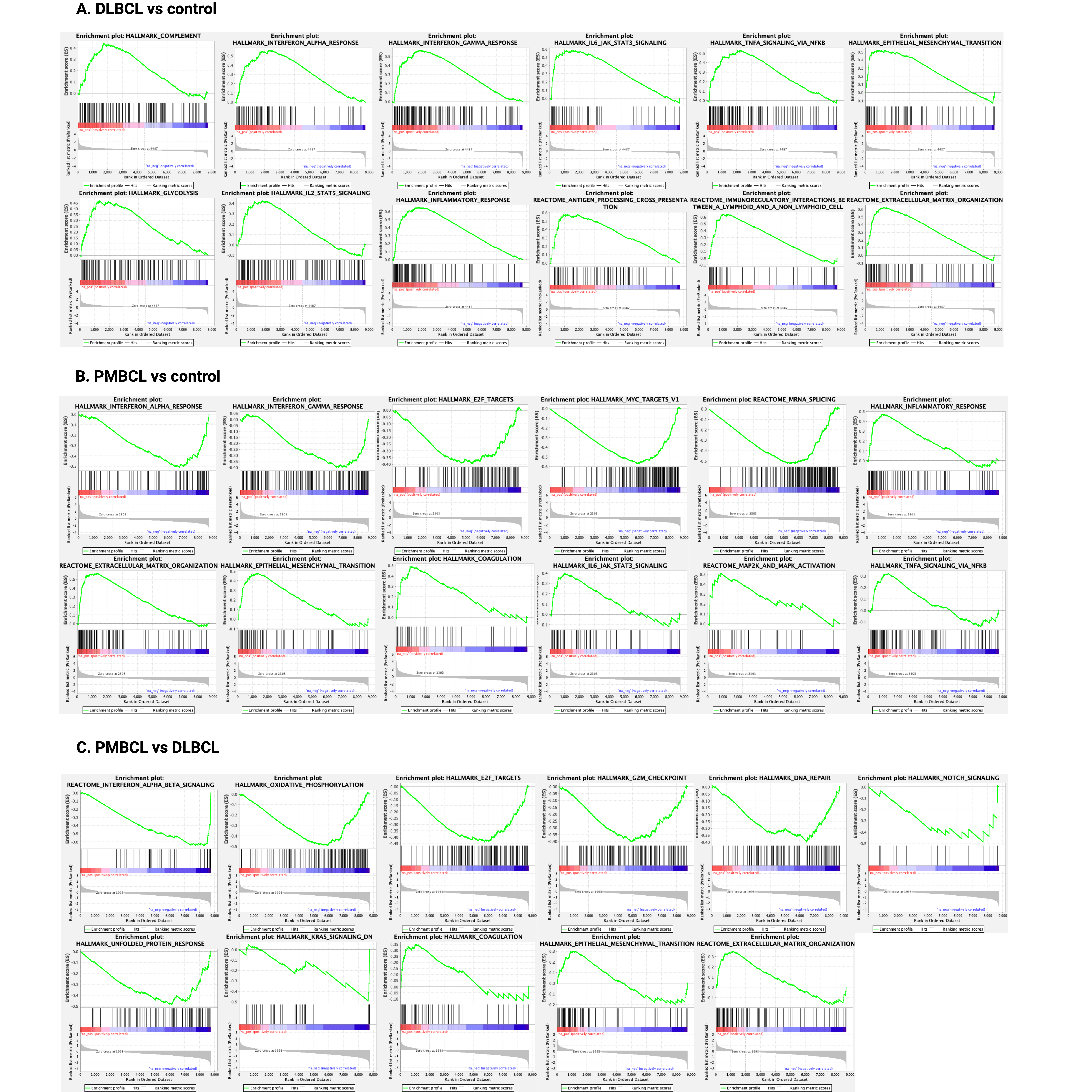

Supplement: Supplementary Figure 1 — Overview of the blood plasma samples included per time point in the study (total of 168 samples). Response at each timepoint was assessed by PET-CT. CR: complete remission; PD: progressive disease; PET-CT: positron emission tomography/computerized tomography. [file DataSheet_1.zip › Supplementary_Figure6.png]

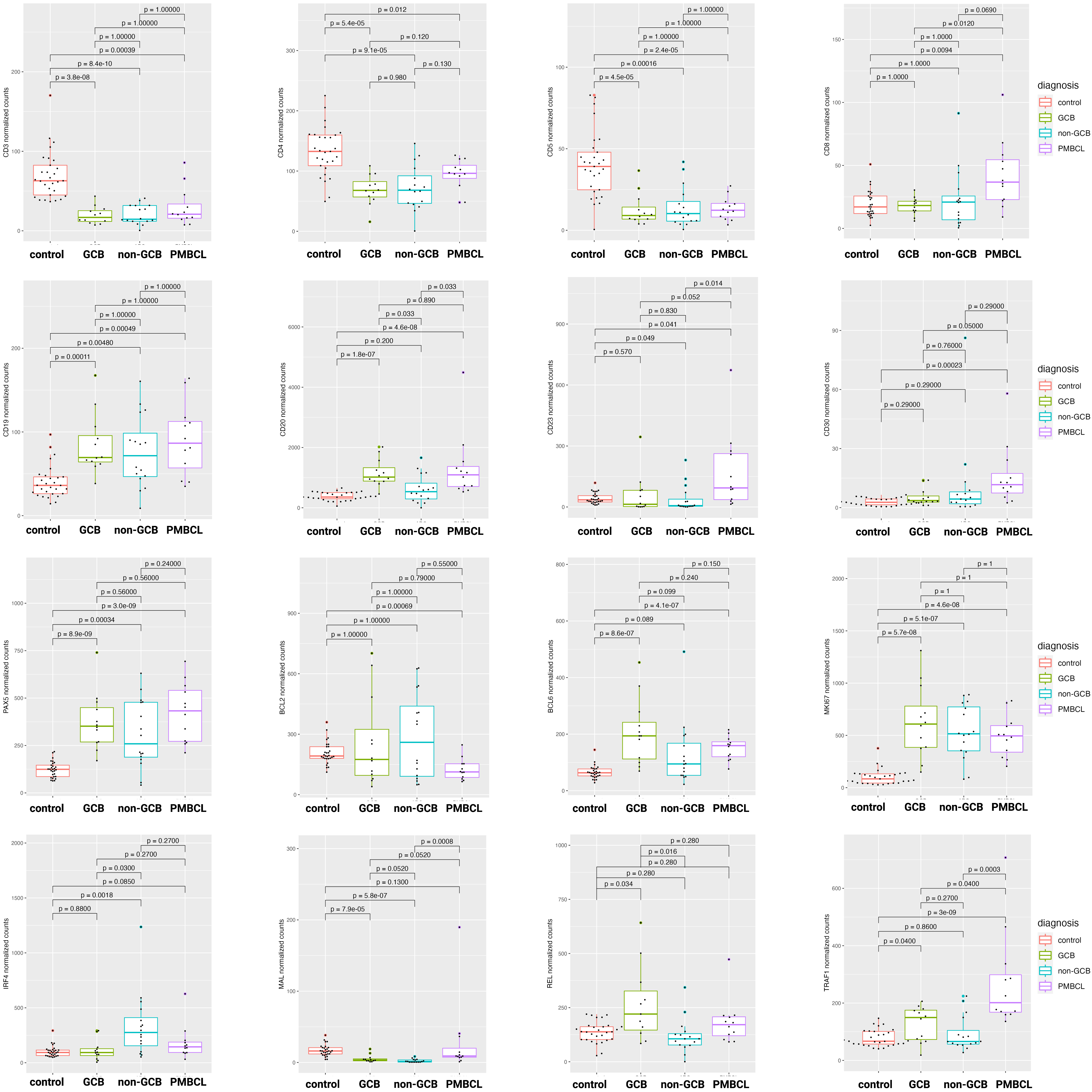

Supplement: Supplementary Figure 1 — Overview of the blood plasma samples included per time point in the study (total of 168 samples). Response at each timepoint was assessed by PET-CT. CR: complete remission; PD: progressive disease; PET-CT: positron emission tomography/computerized tomography. [file DataSheet_1.zip › Supplementary_Figure7.png]

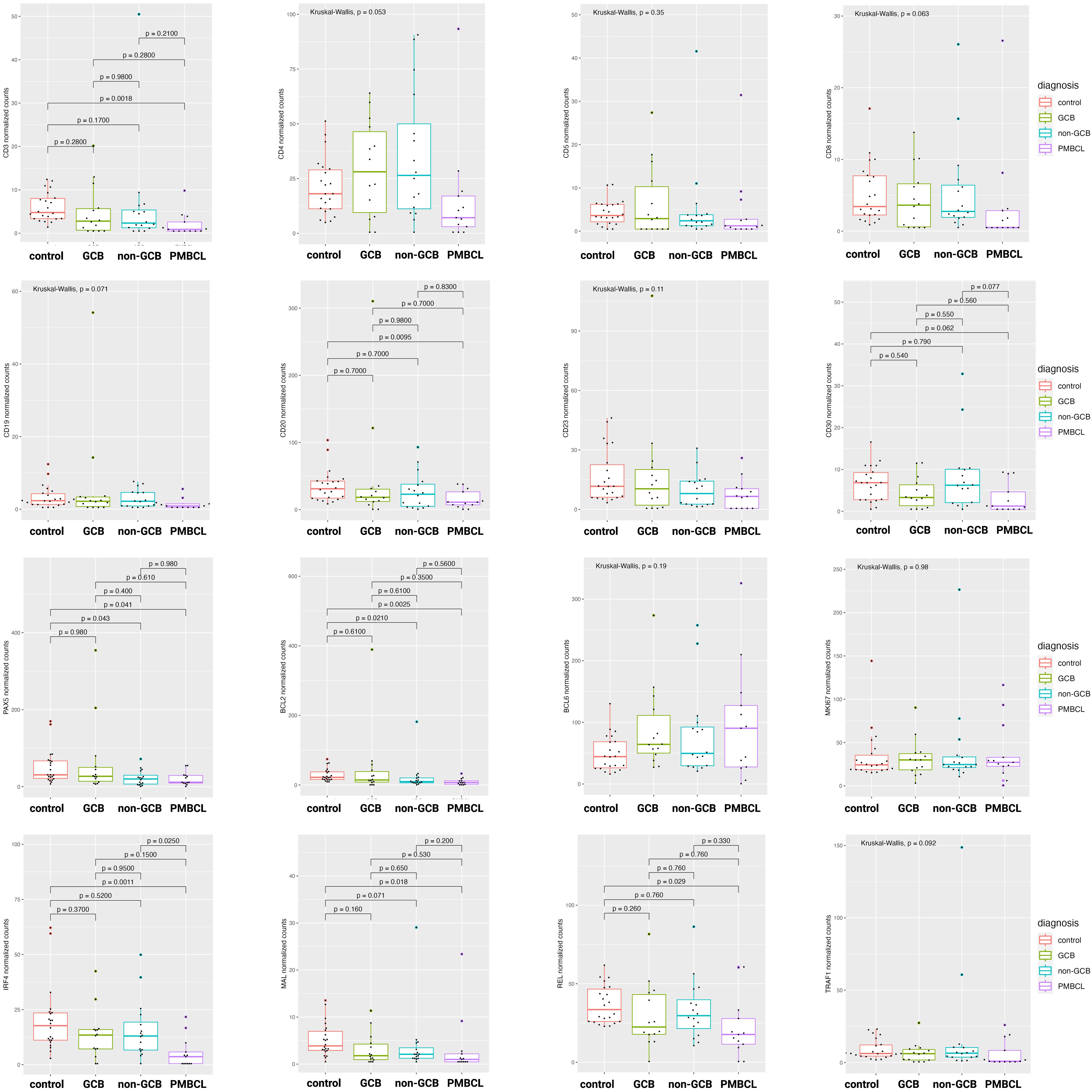

Supplement: Supplementary Figure 1 — Overview of the blood plasma samples included per time point in the study (total of 168 samples). Response at each timepoint was assessed by PET-CT. CR: complete remission; PD: progressive disease; PET-CT: positron emission tomography/computerized tomography. [file DataSheet_1.zip › Supplementary_Figure8.png]

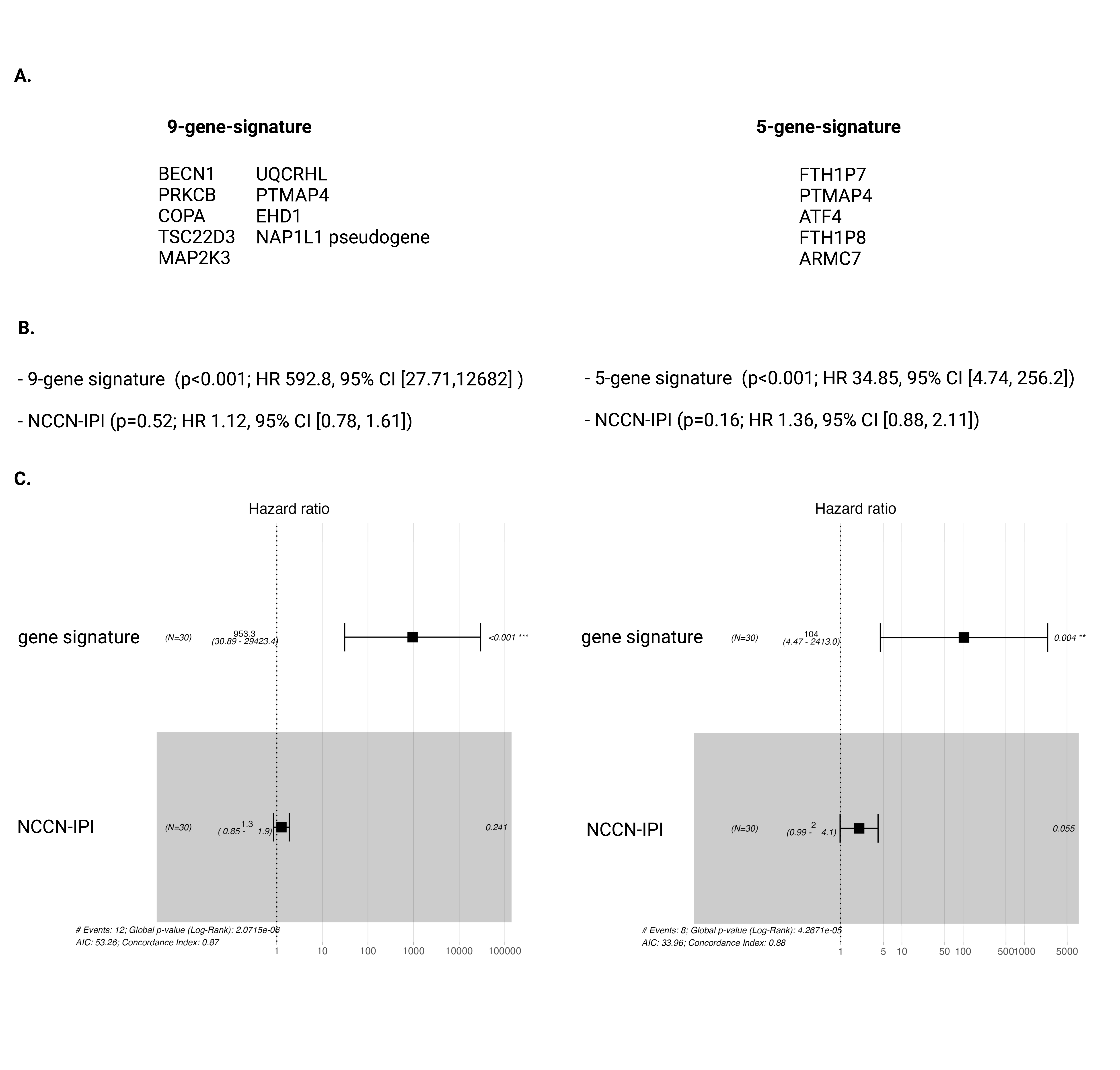

Supplement: Supplementary Figure 1 — Overview of the blood plasma samples included per time point in the study (total of 168 samples). Response at each timepoint was assessed by PET-CT. CR: complete remission; PD: progressive disease; PET-CT: positron emission tomography/computerized tomography. [file DataSheet_1.zip › Supplementary_Figure9.png]
